# Intermittent Fasting Reduces Taurine Availability and Promotes Antitumor Immunity Through PERK Attenuation‐Associated Paraptosis‐Like Cell Death in Colorectal Cancer

**DOI:** 10.1002/advs.78107

**Published:** 2026-10-06

**Authors:** Baohui Song, Sikei Kam, Jingyi Liu, Rui Lv, Xucheng Huo, Zhanghan Chen, Yuelun Dong, Shilun Cai, Bing Li, Ruobing Ren, Yunshi Zhong, Mingyan Cai

**Affiliations:** ^1^ Endoscopy Center Zhongshan Hospital Fudan University Shanghai Digestive Endoscopy Diagnosis and Treatment Engineering Technology Research Center Shanghai China; ^2^ Endoscopy Center Zhongshan Hospital Xuhui Branch Fudan University Shanghai China; ^3^ Shanghai Key Laboratory of Metabolic Remodeling and Health Institute of Metabolism and Integrative Biology Fudan University Shanghai China

**Keywords:** anti‐tumor effect, colorectal cancer, intermittent fasting, PERK, taurine, tumor microenvironment

## Abstract

Intermittent fasting (IF) exerts antitumor activity in colorectal cancer (CRC), but the tumor‐intrinsic metabolic mechanism linking nutrient restriction to antitumor immunity remains unclear. In this study, IF reduced taurine availability and suppressed tumor growth in a CD^8+^ T cell‐dependent manner. Taurine supplementation attenuated this effect, whereas pharmacological inhibition of taurine uptake partially recapitulated fasting‐associated tumor suppression in preclinical models. Mechanistically, taurine is associated with the endoplasmic reticulum chaperone GRP78 and contributes to the maintenance of PERK abundance under nutrient stress. As a metabolic consequence of IF, taurine restriction impaired PERK stability, suppressed downstream ERO1A signaling, and redirected CRC cells from stress tolerance toward paraptosis‐like immunogenic stress. PERK loss enhanced CD^8+^ T‐cell activation, remodeled the tumor microenvironment toward a less suppressive state, and supported tumor control in combination with anti‐PD‐1 therapy. The present study identified taurine restriction as a functional metabolic feature of IF that compromises PERK‐dependent stress adaptation and promotes antitumor immunity in CRC.

## Graphical abstract

This study identifies reduced taurine availability as a mediator of intermittent fasting in colorectal cancer and shows that taurine interacts with GRP78 to maintain PERK abundance post‐transcriptionally. It further shows that taurine depletion destabilizes PERK, induces immunogenic paraptosis, remodels the tumor microenvironment toward a CD8^+^ T cell‐inflamed state, and enhances the efficacy of PD‐1 blockade. Pharmacological inhibition of taurine uptake partially recapitulated taurine‐restriction‐associated effects.

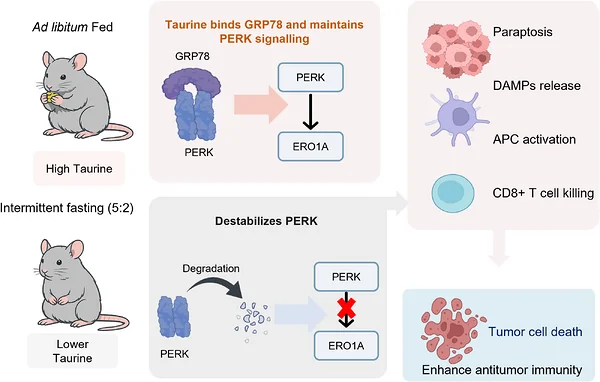

## 1 Introduction

Nutritional interventions are increasingly recognized as biologically relevant modifiers of colorectal cancer (CRC) progression [1]. In addition to reducing caloric intake, dietary interventions can modify systemic metabolism, nutrient availability, and microbiota‐derived metabolites, thereby influencing tumor growth, tumor evolution, and therapeutic response [2]. In preclinical studies, fasting‐related and diet‐based interventions have been associated with improved cancer suppression [3, 4, 5]. Among these strategies, intermittent fasting (IF) has attracted particular interest because it induces coordinated metabolic and endocrine adaptations while preserving periodic refeeding [6]. In several types of cancer, including breast cancer [7, 8], hepatocellular carcinoma [9], and glioblastoma [10], fasting‐related interventions have been associated with tumor regression and improved therapeutic efficacy. Similar effects have also been reported in CRC [11], an immunologically cold tumor type in which most tumors respond poorly to immune checkpoint blockade, in which IF has been demonstrated to enhance anti‐tumor activity and restrain tumor progression. However, the tumor‐cell‐intrinsic mechanism by which nutrient restriction induces these immune‐relevant changes in CRC remains poorly defined.

Taurine, a sulfur‐containing metabolite, is critical for osmotic regulation, redox equilibrium, and cellular stress adaptation. Its intracellular availability is regulated by the taurine transporter SLC6A6, which has been implicated in tumor cell survival, stem‐like properties, and therapeutic resistance in gastrointestinal malignancies [11]. Studies have demonstrated the correlation between taurine‐related pathways and cellular adaptation to metabolic stress [12, 13]. This finding suggests the possibility that taurine may influence the ability of tumor cells to maintain fitness in hostile microenvironmental conditions. Consequently, taurine emerges as a promising mediator that can influence nutrient status and potentially affect tumor behavior.

In this study, reduced taurine availability was identified as a functional metabolic consequence of IF in CRC. Taurine restriction impaired GRP78‐associated PERK maintenance, suppressed downstream stress adaptation, and redirected tumor cells toward paraptosis‐like immunogenic stress, thereby enhancing antitumor immunity and supporting tumor control during PD‐1 blockade.

## 2 Results

### 2.1 Intermittent Fasting Suppressed Colorectal Tumor Growth Through CD^8+^ T‐Cell‐Dependent Antitumor Immunity

The effect of intermittent fasting (IF) was first evaluated in the spontaneous Apc^Min/+^ model using a 5:2 regimen initiated at 6 weeks of age (Figure 1A). IF reduced body‐weight gain relative to ad libitum (AL) feeding (Figure 1B). At the end of the intervention, the IF‐treated group exhibited a lower intestinal tumor burden, characterized by reductions in both tumor number and diameter (Figure 1C–E). Tumor proliferation was inhibited in the IF‐treated group, as shown by decreased Ki‐67 staining in tumor tissues (Figure 1F,G). Immunofluorescence revealed increased intratumoral CD^3+^CD^8+^ T‐cell infiltration in IF‐treated mice (Figure 1G).

**FIGURE 1:**
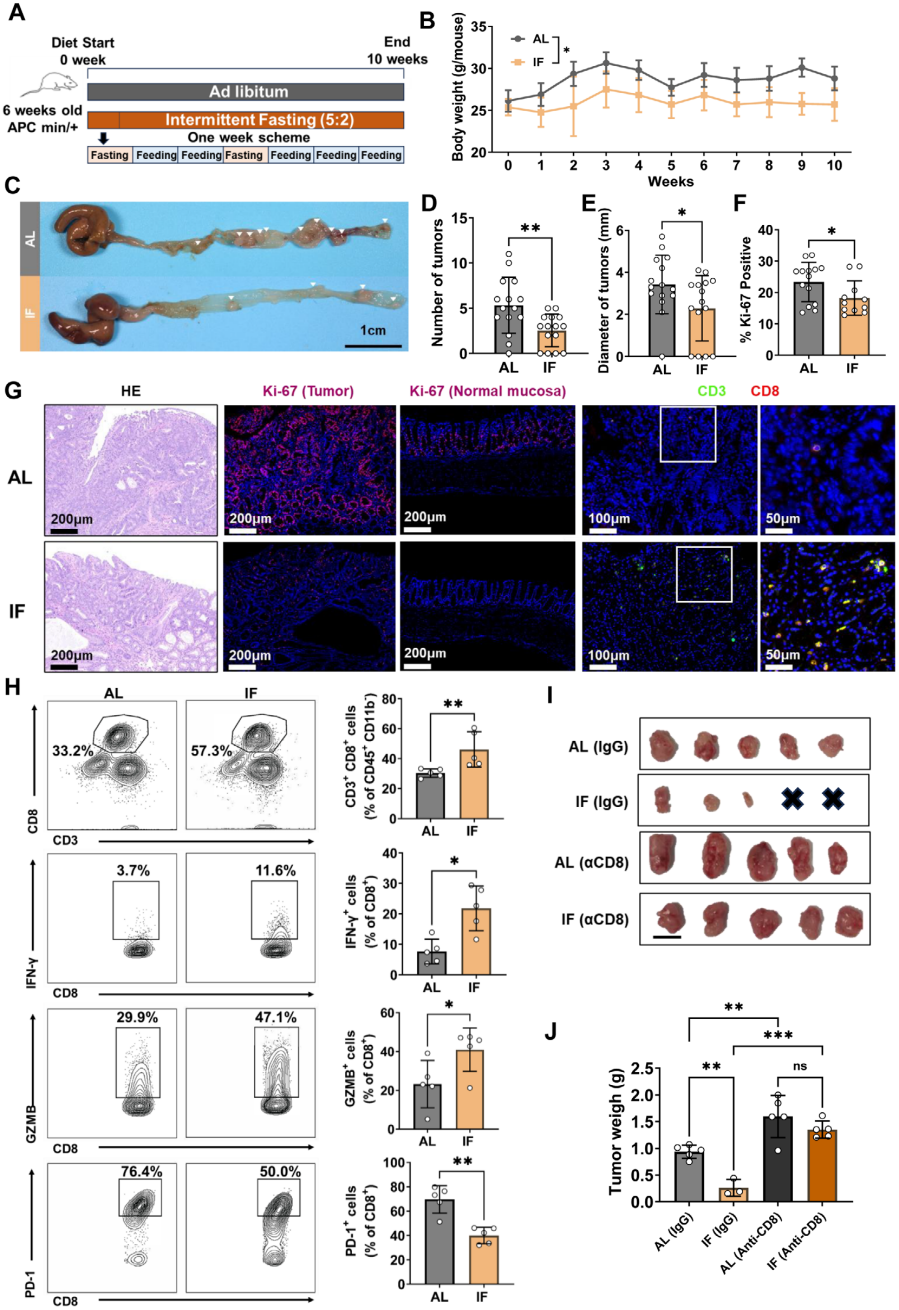
Intermittent fasting suppresses intestinal tumor growth and enhances CD8^+^ T‐cell‐mediated antitumor immunity. (A) Schematic of the 5:2 intermittent fasting (IF) regimen in Apc^min/+^ mice. Mice were maintained under ad libitum feeding (AL) or IF until endpoint analysis. (B) Body weight during dietary intervention. *n* = 11–15 per group.Two‐way ANOVA. (C) Representative images of intestinal tumors from AL‐ and IF‐fed mice. (D–F) Quantification of tumor number (D), tumor diameter (E), and Ki‐67‐positive tumor cells (F). *n* = 11–15 per group. Unpaired t‐test. (G) Representative H&E staining, Ki‐67 staining, and CD8 immunostaining/immunofluorescence in intestinal tumor tissues from AL‐ and IF‐fed mice. (H) Representative flow‐cytometry plots and quantification of tumor‐infiltrating CD8^+^ T cells and effector/exhaustion markers, including IFN‐γ, GZMB, and PD‐1. *n* = 5 per group. Unpaired t‐test. (I, J) Representative tumor images (I) and tumor weight (J) from AL‐ or IF‐fed mice treated with control IgG or anti‐CD8 antibody. *n* = 3–5/group. One‐way ANOVA. Scale bars as indicated. Data are presented as mean ± SD. ns, not significant; ^*^p < 0.05, ^**^ *p* < 0.01, ^***^ *p* < 0.001.

We next validated this phenotype in a syngeneic MC38 tumor model. IF markedly suppressed MC38 tumor growth and reduced final tumor size (Figure S1A–C). Immunofluorescence showed increased CD^3+^ and CD^8+^ T‐cell infiltration at the tumor margin and within the tumor core, whereas Ki‐67 staining was reduced in tumors from IF‐fed mice (Figure S1D). Tumor interstitial fluid (TIF) was collected to assess extracellular soluble immunogenic stress‐related signals within the tumor microenvironment. IF also increased SEC61β expression in tumor tissue and elevated HMGB1 and IFN‐β levels in TIF (Figure S1E–G).

Flow‐cytometric profiling further demonstrated that IF reshaped the tumor immune microenvironment. The proportion of CD3^+^CD8^+^ T cells was increased in tumors from IF‐fed mice, accompanied by higher frequencies of IFN‐γ^+^ and GZMB^+^ cells and a lower proportion of PD‐1^+^ cells in CD8^+^ T cells (Figure 1H, Figure S1H). These changes suggested that IF increased CD8^+^ T‐cell accumulation and also enhanced their effector status. To test whether the CD8^+^ T cells were required for the antitumor effect of IF, the anti‐CD8 antibody was used in the syngeneic model. Anti‐CD8 largely reversed the tumor‐suppressive effect of IF, suggesting that CD8^+^ T cells are required for IF‐mediated tumor control (Figure 1I,J).

### 2.2 IF‐Associated Taurine Restriction Contributes Antitumor Immunity

To identify IF‐regulated metabolites that may contribute to IF‐mediated tumor suppression, serum metabolomic profiling was performed in AL‐ and IF‐fed Apc^Min/+^ mice. Metabolomics analyses confirmed clear metabolic separation between the AL and IF groups (Figure S2A–C). Taurine was among the most prominently reduced metabolites after IF (Figure 2A). Taurine quantification further validated this reduction in both serum and tumor tissues (Figure 2B,C). Exogenous taurine enhanced DLD1 cell proliferation with an EC50 of 11.21 µm (Figure S2D).

**FIGURE 2:**
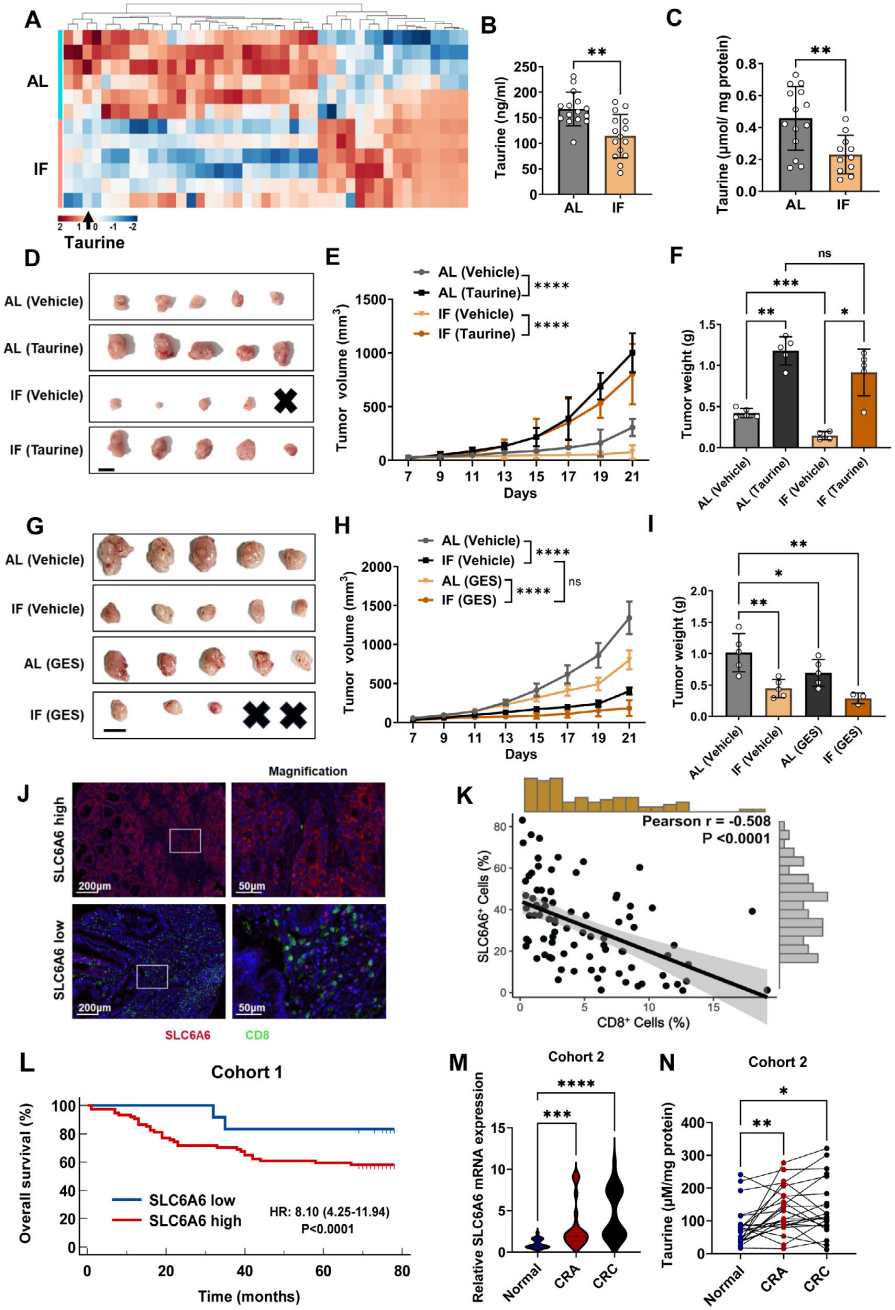
Taurine restriction mediates the antitumor effect of intermittent fasting. (A) Heatmap of serum metabolomic profiling highlighting metabolites altered by IF in Apc^min/+^ mice, including taurine. *n* = 6 per group. (B, C) Taurine concentrations in mouse serum and tumor tissue under AL and IF conditions in Apc^min/+^ mice. *n* = 11–15 per group. Unpaired *t*‐test. (D–F) Representative tumor images (D), tumor growth curves (E), and tumor weight quantification (F) from AL‐ or IF‐fed mice treated with vehicle or taurine supplementation. *n* = 4–5 per group. Two‐way ANOVA for tumor growth curves; one‐way ANOVA for tumor weight. (G–I) Representative tumor images (G), tumor growth curves (H), and tumor weight quantification (I) from AL‐ or IF‐fed mice treated with vehicle or the taurine‐transporter inhibitor GES. *n* = 3–5 per group. Two‐way ANOVA for tumor growth curves; one‐way ANOVA for tumor weight. (J) Representative multiplex immunofluorescence images showing SLC6A6 and CD8 expression in human CRC tissues. (K) Correlation between SLC6A6 positivity and CD8^+^ T‐cell abundance in human CRC tissues. *n* = 85 cases. Pearson correlation analysis. (L) Kaplan‐Meier analysis of overall survival according to SLC6A6 expression. *n* = 85 patients. Log‐rank test. (M) SLC6A6 mRNA expression in normal mucosa, colorectal adenoma (CRA), and CRC tissues from two independent cohorts. *n* = 23 per group. One‐way ANOVA. (N) Taurine abundance in TIF collected from matched normal mucosa, colorectal adenoma (CRA), and CRC tissues. *n* = 23 matched cases. Repeated‐measures one‐way ANOVA. Scale bars are shown in the corresponding panels. Data are presented as mean ± SD unless otherwise indicated. *ns, not significant*; ^*^ *p* < 0.05, ^**^ *p* < 0.01, ^****^ *p* < 0.0001.

The relationship between systemic taurine metabolism and local tumor taurine exposure was then examined across multiple compartments. Because the liver is a major site of taurine biosynthesis, hepatic cysteine dioxygenase 1 (CDO1), a key enzyme involved in cysteine oxidation upstream of taurine production [14], was measured. IF reduced hepatic CDO1 expression, suggesting decreased systemic taurine biosynthesis (Figure S2E). Taurine levels were also reduced in serum, tumor interstitial fluid (TIF), and tumor tissues after IF (Figure S2F–H). These results indicated that IF establishes a systemic taurine‐restricted state that extends to the tumor microenvironment and reduces intratumoral taurine abundance.

The functional relevance of taurine reduction was further tested by taurine supplementation during IF. Taurine supplementation largely reversed IF‐mediated suppression of tumor growth and final tumor weight (Figure 2D–F), indicating that taurine depletion is a functional mediator of IF‐induced tumor suppression. Conversely, pharmacological inhibition of taurine uptake with the taurine transporter inhibitor guanidinoethyl sulfonate (GES) suppressed tumor growth under AL conditions (Figure 2G–I). GES reduced tumor taurine accumulation without reproducing the broader systemic taurine reduction induced by IF (Figure S2F–H). Multiplex immunofluorescence further showed that taurine supplementation increased CD^8+^ T‐cell infiltration under AL conditions and was accompanied by greater PD‐1/TIM‐3 co‐expression, while under IF conditions it increased the PD‐^1+^TIM‐^3+^ fraction among CD8^+^ cells without reducing overall CD8^+^ T‐cell infiltration. In contrast, GES increased CD8^+^ cell infiltration under both AL and IF conditions, consistent with its enhanced tumor‐suppressive effect (Figure S2I,J).

SLC6A6 expression is a marker of taurine uptake capacity [15]. To further examine the clinical relevance of taurine uptake in CRC, we used a CRC follow‐up cohort (Cohort 1) and an adenoma‐carcinoma sequence cohort (Cohort 2). In cohort 1 (Table S1), 85 surgically resected patients with survival follow‐up were included; SLC6A6 expression was analyzed in relation to CD8^+^ T‐cell infiltration and overall survival. Multiplex immunofluorescence showed that tumor tissue with high SLC6A6 expression contained fewer CD8^+^ T cells (Figure 2J), and quantitative analysis confirmed a negative correlation between SLC6A6 positivity and CD8^+^ T‐cell abundance (Figure 2K). Clinically, high SLC6A6 expression was associated with poorer overall survival, and SLC6A6 expression increased during progression from normal mucosa to colorectal adenoma and carcinoma (Figure 2L,M). In cohort 2 (Table S2), direct taurine measurements in matched human tissue samples further showed increased taurine abundance in TIF during colorectal tumorigenesis (Figure 2N). These findings were corroborated by public CRC datasets, which demonstrated that higher SLC6A6 expression was associated with poorer survival outcomes and negatively correlated with CD8^+^ T‐cell infiltration rate (Figure S3A–H).

### 2.3 Taurine‐Associated GRP78 Contributes to PERK Protein Stability Under Nutrient Stress

Metabolites can function as signaling molecules through interactions with specific protein targets [16]. Taurine has been implicated in diverse signaling events, yet the molecular targets underlying these activities remain incompletely defined [12, 17, 18]. To define the mediator of taurine, the drug affinity responsive target stability (DARTS) assay was performed. HSPA5 (GRP78) was identified as the dominant candidate in this screen, exhibiting the highest unique sequence coverage (46.9%) among the recovered proteins (Figure 3A). This association was independently supported by a dose‐dependent protease protection assay, in which increasing taurine concentrations (0–15 µm) progressively preserved GRP78 abundance after protease treatment (Figure 3B). Biotinylated taurine pulldown supported GRP78 enrichment, and this enrichment was reduced by excess unlabeled taurine (Figure 3C).

**FIGURE 3:**
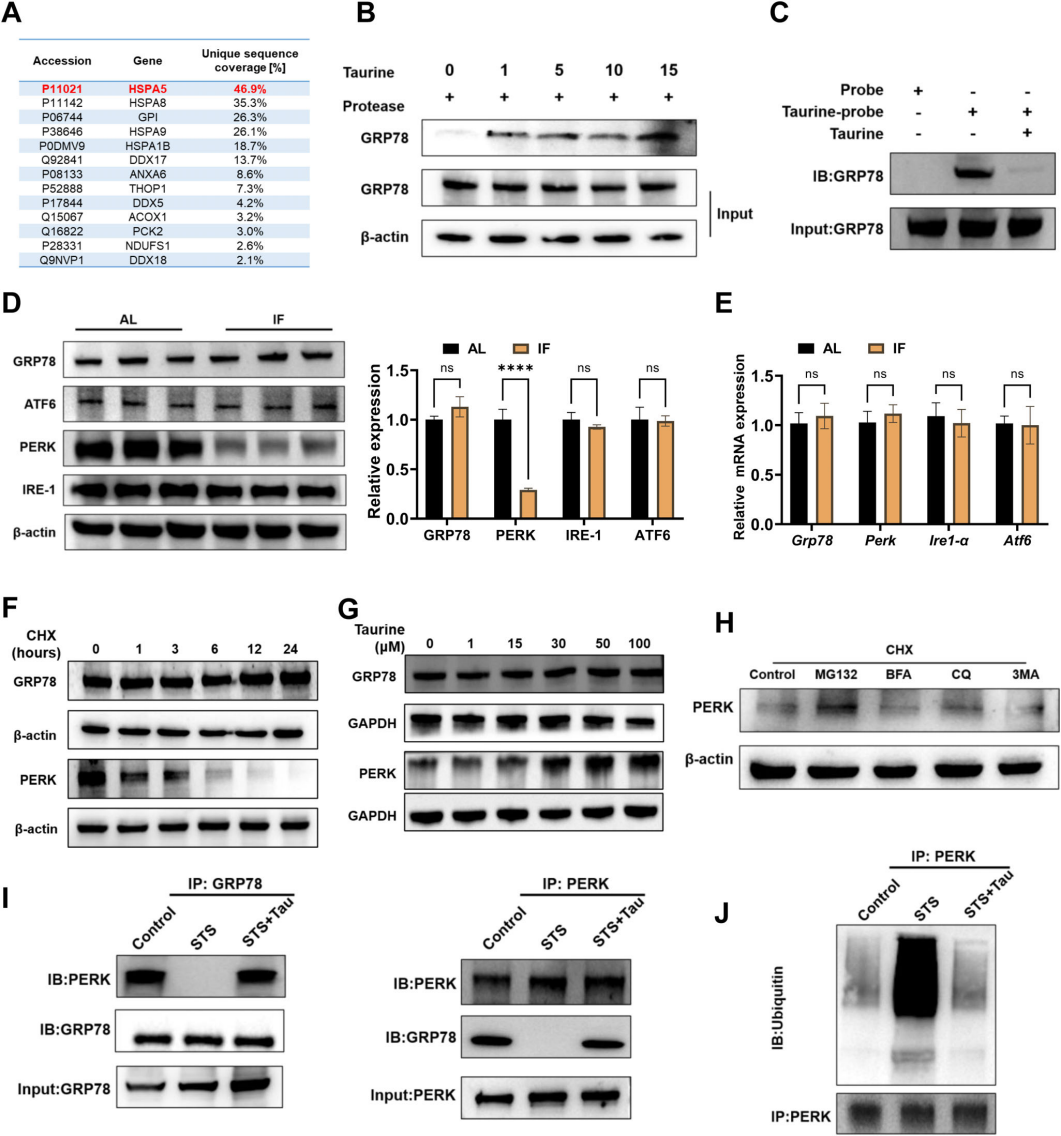
Taurine associates with GRP78 and maintains PERK protein stability under stress. (A) DARTS‐MS‐based proteomic screening of taurine‐associated proteins, identifying GRP78/HSPA5 as a candidate taurine‐associated protein. (B) Protease‐protection assay showing dose‐dependent protection of GRP78 by taurine. Representative blots from 3 independent experiments are shown. (C) Biotinylated taurine pulldown showed GRP78 enrichment and competition by excess free taurine. Representative blots from 3 independent experiments are shown. (D) Western blot analysis and quantification of GRP78, PERK, IRE1α, and ATF6 protein expression in tumor tissues from AL‐ and IF‐fed mice. *n* = 3 per group. Unpaired t‐test. (E) Quantitative PCR analysis of *Grp78, Perk, Ire1α*, and *Atf6* mRNA expression in AL‐ and IF‐fed tumor tissue. *n* = 3 per group. Unpaired t‐test. (F) Cycloheximide chase analysis of GRP78 and PERK protein abundance under stress. Representative blots from 3 independent experiments are shown. (G) Western blot analysis of GRP78 and PERK after treatment with increasing concentrations of taurine. Representative blots from 3 independent experiments are shown. (H) Effects of MG132, brefeldin A (BFA), chloroquine (CQ), and 3‐methyladenine (3‐MA) on PERK protein abundance under stress. Representative blots from 3 independent experiments are shown. (I) Co‐immunoprecipitation analysis of the GRP78‐PERK interaction under control, STS, and STS plus taurine conditions. Representative blots from 3 independent experiments are shown. (J) PERK ubiquitination after stress treatment with or without taurine supplementation. Representative blots from 3 independent experiments are shown. Data are presented as mean ± SD. ns, not significant; ^****^p < 0.0001.

GRP78 is a central ER‐resident chaperone and upstream regulator of unfolded protein response (UPR) sensors [19]. GRP78‐regulated UPR sensors were then examined in tumors from AL‐ and IF‐fed mice. IF selectively reduced PERK protein abundance, whereas GRP78, IRE1α, and ATF6 protein levels were not significantly changed (Figure 3D). The mRNA levels of *Grp78, Perk, Ire1α*, and *Atf6* were not significantly altered by IF (Figure 3E), suggesting that fasting regulates PERK mainly at the protein level. To further examine PERK protein stability under nutrient‐stress conditions, short‐term starvation (STS), an established in vitro nutrient‐stress model [8], was used. In cycloheximide (CHX) chase experiments performed under STS, PERK protein abundance declined after inhibition of de novo protein synthesis. In contrast, taurine supplementation preserved PERK expression in a concentration‐dependent manner without substantially altering GRP78 expression (Figure 3F,G). Consistently, taurine did not alter GRP78 or PERK mRNA expression under STS (Figure S4A,B). Under STS and in the presence of CHX, pharmacological inhibition experiments showed that MG132 partially restored PERK protein abundance. In contrast, other inhibitors had limited effects, suggesting that STS‐induced PERK loss is mediated predominantly through proteasome‐dependent degradation (Figure 3H). The involvement of GRP78 in taurine‐mediated preservation of PERK was therefore further examined. Moreover, STS weakened the GRP78‐PERK interaction, whereas taurine restored GRP78 association with PERK (Figure 3I). STS also increased PERK ubiquitination, which was attenuated by taurine supplementation (Figure 3J). These findings support a GRP78‐associated mechanism by which taurine contributes to PERK protein maintenance under STS, at least in part by limiting ubiquitination‐associated PERK degradation.

### 2.4 PERK Loss Induces Paraptosis‐Like Tumor‐Cell Stress and Immunogenic Signaling

To determine how PERK loss influences tumor cell fate under nutrient stress, PERK‐knockdown (KD) DLD1 cells were exposed to STS (Figure S5A). PERK‐KD reduced tumor‐cell proliferation under STS, as shown by CCK‐8 and EdU assays (Figure 4A,B, Figure S5B). Pharmacological inhibitor screening under STS showed that inhibitors targeting apoptosis, necroptosis, ferroptosis, pyroptosis, autophagy, or PARP‐dependent death produced limited or incomplete protection, whereas CHX produced the most prominent reduction in PERK KD‐associated cell death (Figure 4C). This observation was further examined in PERK WT, PERK KD, and PERK KD plus taurine‐treated cells, in which CHX attenuated the increase in Annexin V positivity induced by PERK depletion.

**FIGURE 4:**
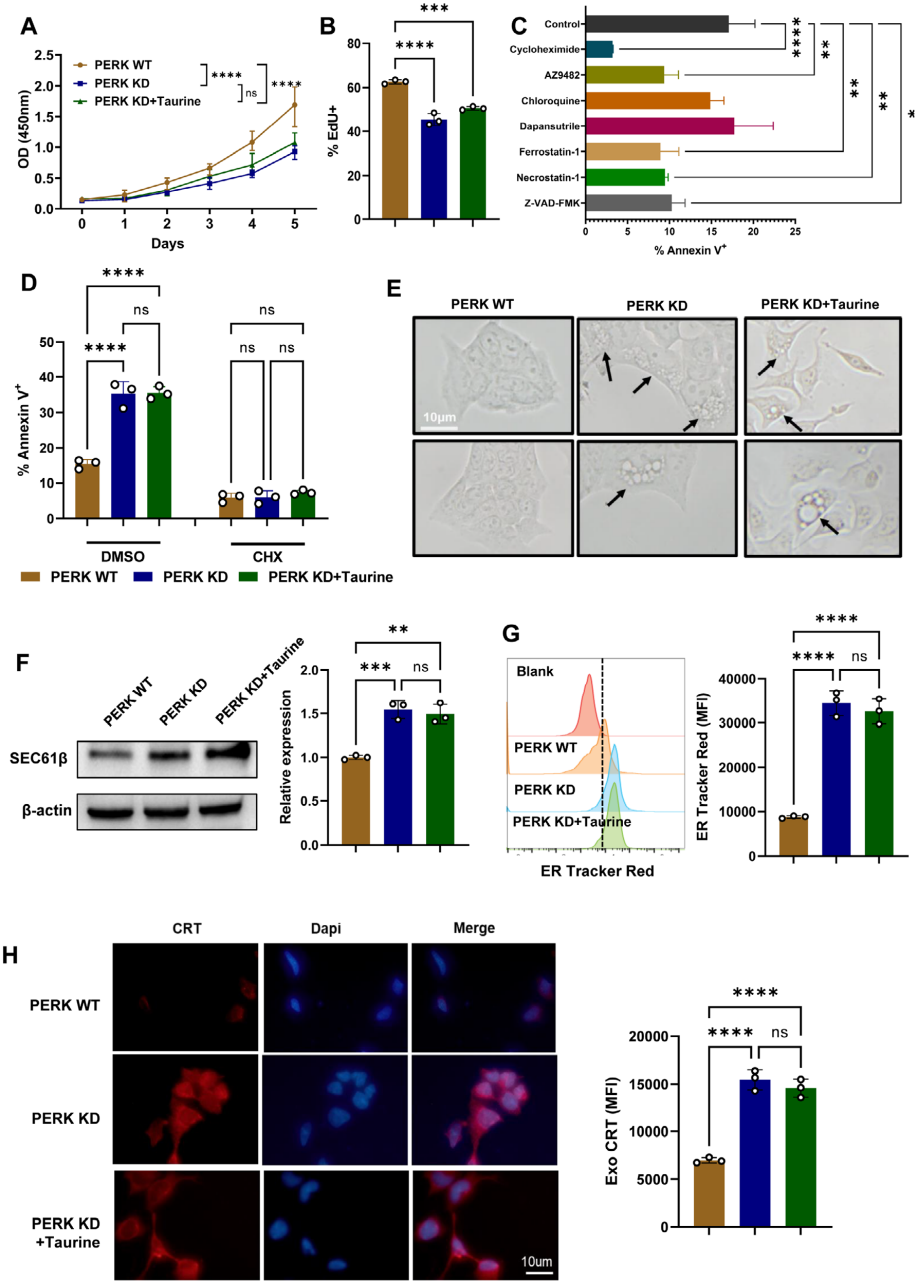
PERK depletion induces paraptosis‐like cell death and immunogenic signaling in CRC cells. (A) Cell proliferation of PERK WT, PERK KD, and PERK KD plus taurine‐treated CRC cells exposed to STS, and assessed by CCK‐8 assay. *n* = 5 independent experiments. Two‐way ANOVA. (B) EdU incorporation in PERK WT, PERK KD, and PERK KD plus taurine‐treated CRC cells exposed to STS. *n* = 3 independent experiments. One‐way ANOVA. (C) Pharmacological inhibitor screening of cell death when exposed to STS using inhibitors targeting apoptosis, necroptosis, ferroptosis, pyroptosis, autophagy, and protein synthesis. *n* = 3 independent experiments. One‐way ANOVA. (D) Annexin V‐positive cells after DMSO or cycloheximide (CHX) treatment in PERK WT, PERK KD, and PERK KD plus taurine‐treated cells exposed to STS. *n* = 3 independent experiments. Two‐way ANOVA. (E) Representative bright‐field images showing cytoplasmic vacuolization in PERK KD cells under STS. Arrows indicate vacuolated cells. (F) Western blot analysis and quantification of SEC61β expression. *n* = 3 independent experiments. One‐way ANOVA. (G) Representative ER‐Tracker Red fluorescence histograms and quantification of ER expansion. *n* = 3 independent experiments. One‐way ANOVA. (H) Representative immunofluorescence images and quantification of cell‐surface calreticulin (CRT). *n* = 3 independent experiments. One‐way ANOVA. Scale bars are shown in the corresponding panels. Data are presented as mean ± SD. *ns, not significant*; ^*^ *p* < 0.05, ^**^ *p* < 0.01,^***^ *p* < 0.001, ^****^ *p* < 0.0001.

In contrast, taurine supplementation did not reverse this phenotype (Figure 4D). Morphologically, PERK‐deficient cells developed extensive cytoplasmic vacuolization under STS, and this change was not reversed by taurine supplementation (Figure 4E). Consistently, PERK depletion increased SEC61β expression and ER‐Tracker fluorescence, both of which remained elevated despite taurine supplementation (Figure 4F,G). Taurine supplementation failed to reverse these changes after PERK KD.

When exposed to STS, PERK KD also enhanced multiple immunogenic cell‐stress signals, including increased cell‐surface calreticulin (CRT) exposure, elevated HMGB1 release, and increased IFN‐β production, when co‐cultured with PBMCs (Figure 4H; Figure S5C–E). PERK‐deficient cells also exhibited enhanced susceptibility to PBMC‐mediated killing (Figure S5F). PERK KD tumor cells increased *Ifnb1, Ifit3, and Isg15* expression in co‐cultured PBMCs, indicating activation of type I interferon‐associated signaling (Figure S5G–I). These results indicated that PERK KD induced paraptosis‐like cell death and promoted immunogenic signaling.

### 2.5 PERK Deficiency Suppressed Tumor Growth and Promoted Antitumor Immunity In Vivo

We then tested whether PERK loss could reproduce the antitumor immune phenotype observed under IF in vivo. In syngeneic tumor models, PERK knockdown suppressed tumor growth (Figure 5A–C). In PERK‐deficient tumors, taurine supplementation did not rescue tumor growth, indicating that the tumor‐cell‐intrinsic growth‐supporting effect of taurine requires intact PERK. The residual systemic effects of taurine may reflect PERK‐independent or immune‐cell‐related actions in vivo. Immunofluorescence analysis showed that PERK knockdown increased CD3^+^ CD8^+^ T‐cell infiltration and decreased Ki‐67^+^ tumor cells (Figure 5D,E). Flow‐cytometric analysis confirmed that PERK KD increased CD8^+^ T‐cell abundance and enhanced CD8^+^ T‐cell activation, as reflected by increased CD69, GZMB, IFN‐γ, and Ki‐67 expression (Figure 5F, Figure S6A). Flow‐cytometric analysis further showed that PERK depletion remodeled the tumor‐infiltrating myeloid compartment. Within live CD45^+^ CD11b^+^ myeloid cells, PERK KD increased the proportion of antigen‐presenting cDCs (CD11c^+^MHC‐II^+^), supporting enhanced myeloid antigen‐presenting activity. By contrast, PERK KD reduced myeloid‐derived suppressor cells (M‐MDSCs) and granulocytic MDSCs. Macrophage polarization was also shifted toward an inflammatory phenotype, with the increase in M1‐like macrophages while the decrease in M2‐like macrophages in PERK ‐deficient tumors. (Figure 5G; Figure S6B). Because TIF reflects the extracellular fluid compartment within the tumor microenvironment, it was collected to assess local extracellular metabolites and immunogenic stress‐related signals. PERK KD also elevated HMGB1 and IFN‐β in TIF (Figure S6C,D). Taurine supplementation failed to restore these myeloid changes after PERK KD.

**FIGURE 5:**
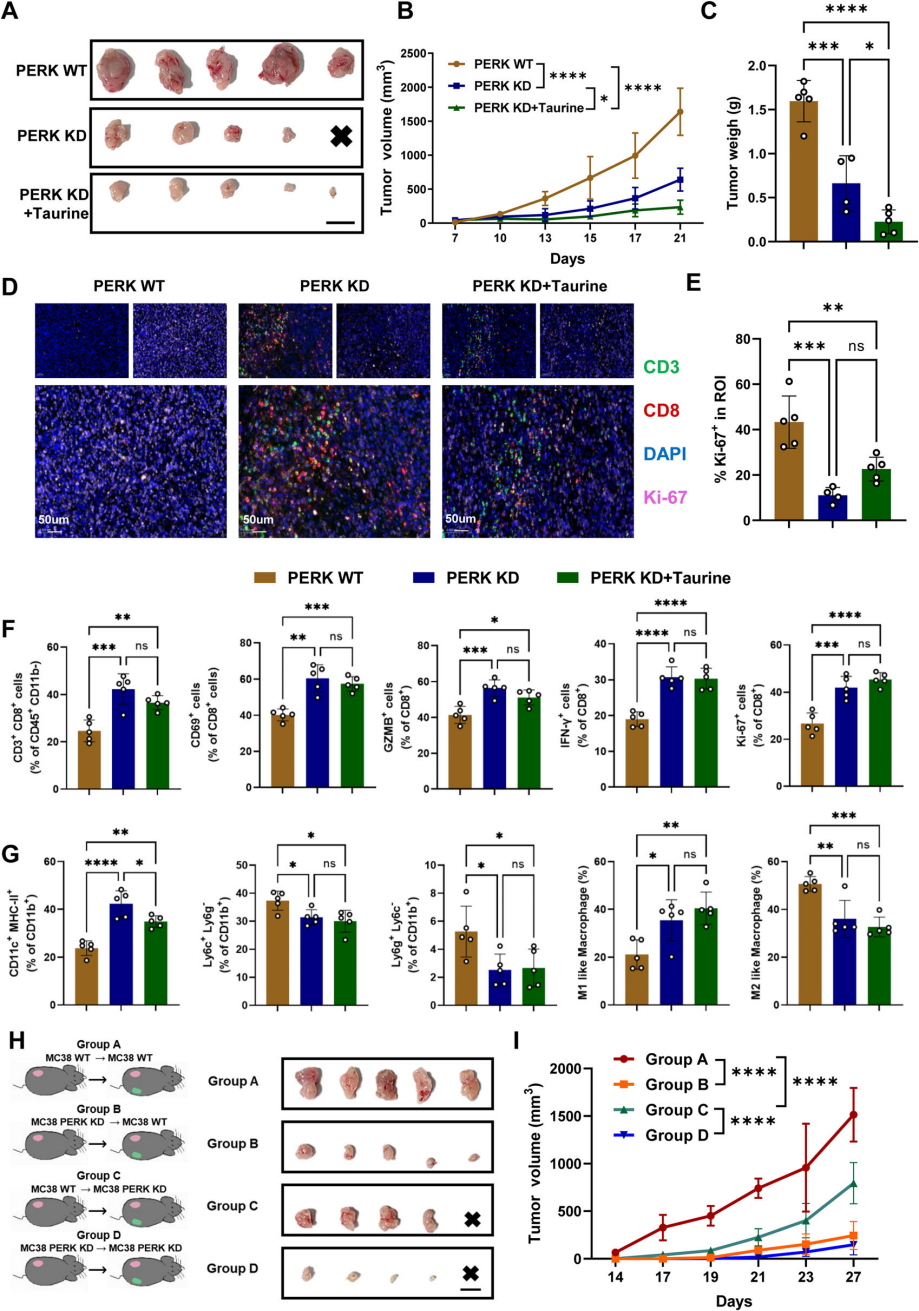
PERK loss suppresses tumor growth and remodels the tumor immune microenvironment. (A–C) Representative tumor images (A), tumor growth curves (B), and tumor weight quantification (C) of PERK WT, PERK KD, and PERK KD plus taurine tumors. *n* = 4–5 per group. Two‐way ANOVA for tumor growth curves; one‐way ANOVA for tumor weight. (D, E) Representative immunofluorescence images of CD3, CD8, and Ki‐67 staining in tumor tissues (D) and quantification (E) of Ki‐67^+^ cells in tumor tissues. *n* = 5 per group. One‐way ANOVA. (F) Flow‐cytometric quantification of CD8^+^ T‐cell infiltration and activation, including CD69^+^, GZMB^+^, IFN‐γ^+^, and Ki‐67^+^ CD8^+^ T cells. *n* = 5 per group. One‐way ANOVA. (G) Flow‐cytometric quantification of tumor‐infiltrating myeloid populations, including cDCs (CD11c^+^MHC‐II^+^), monocytic myeloid cells/M‐MDSCs (Ly6C^+^Ly6G^−^), granulocytic myeloid cells/PMN‐MDSCs (Ly6G^+^Ly6C^−^), M1‐like macrophages, and M2‐like macrophages within the CD11b^+^ myeloid compartment. *n* = 5 per group. One‐way ANOVA. (H) Schematic of the bilateral tumor model used to evaluate local and systemic effects of PERK depletion. Group A served as the control setting with MC38 PERK WT tumors on both sides. Group B tested whether a PERK KD tumor could suppress the growth of a contralateral PERK WT tumor. Group C tested the intrinsic growth of a contralateral PERK KD tumor in the presence of a PERK WT tumor. Group D tested the condition in which both tumors were PERK KD. *n* = 4–5 per group. (I) Representative tumor images and tumor growth curves from bilateral tumor‐bearing mice implanted with PERK WT and/or PERK KD MC38 cells. *n* = 4–5 per group. Two‐way ANOVA. Scale bars are shown in the corresponding panels. Data are presented as mean ± SD. *ns, not significant*; ^*^ *p* < 0.05, ^**^ *p* < 0.01, ^***^ *p* < 0.001, ^****^ *p* < 0.0001.

To assess whether PERK loss was associated with systemic antitumor immunity, the bilateral colorectal tumor model was established in which PERK WT or PERK KD tumor cells were implanted into opposite flanks. PERK‐deficient tumors exhibited impaired growth, and their presence was associated with reduced growth of contralateral tumors, consistent with a systemic immune‐mediated effect (Figure 5H,I).

### 2.6 The PEERO1A Axis Supports Colorectal Tumor Growth and Immune Evasion

To identify downstream transcriptional programs associated with IF‐induced PERK suppression, RNA‐seq analysis was performed in tumors from AL‐ and IF‐fed mice. Differential expression analysis revealed broad transcriptional remodeling after IF (Figure 6A). Gene‐set enrichment analysis showed that the CHOP‐related ER‐stress response program was significantly reduced after IF, accompanied by changes in protein folding, translation, glycolysis, adaptive immune response, CD^8^^+^ T‐cell TNF response, and antigen‐processing pathways (Figure S7A–I). Among CHOP‐associated candidate genes, ERO1A showed the most prominent and consistent reduction after IF, whereas other candidates showed weaker or less consistent changes (Figure S7B,C). These transcriptomic findings suggested ERO1A as a fasting‐suppressed CHOP‐associated ER‐stress gene.

**FIGURE 6:**
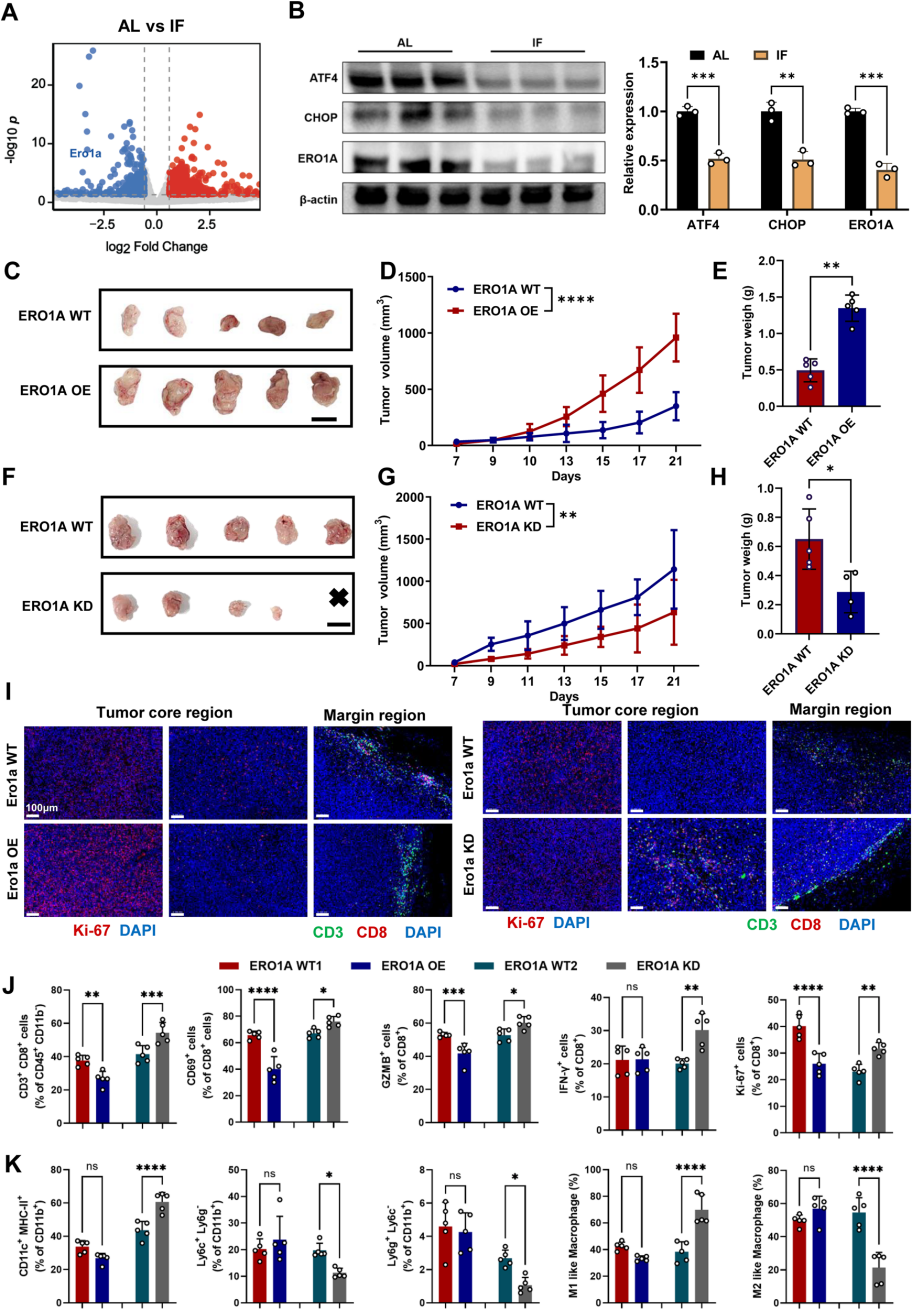
ERO1A acts downstream of the PERK‐associated stress‐adaptation axis to promote tumor growth and immune evasion. (A) Volcano plot showing differentially expressed genes in tumors from AL‐ and IF‐fed mice. *n* = 3 per group. (B) Western blot analysis and quantification of ATF4, CHOP, and ERO1A protein expression in AL‐ and IF‐fed tumors. *n* = 3 per group. Unpaired *t*‐test. (C–E) Representative tumor images (C), tumor growth curves (D), and tumor weight quantification (E) of ERO1A WT and ERO1A‐overexpressing tumors. *n* = 4–5 per group. Two‐way ANOVA for tumor growth curves; unpaired *t*‐test for tumor weight. (F–H) Representative tumor images (F), tumor growth curves (G), and tumor weight quantification (H) of ERO1A WT and ERO1A‐knockdown tumors. *n* = 4–5 per group. Two‐way ANOVA for tumor growth curves; unpaired *t*‐test for tumor weight. (I) Representative immunofluorescence images showing Ki‐67, CD3, and CD8 staining in ERO1A WT, ERO1A‐overexpressing, and ERO1A‐knockdown tumors. (J) Flow‐cytometric quantification of CD8^+^ T‐cell activation and tumor‐infiltrating myeloid populations in ERO1A WT, ERO1A‐overexpressing, and ERO1A‐knockdown tumors. *n* = 5 per group. Unpaired t‐test for two‐group comparisons. (K) Flow‐cytometric quantification of tumor‐infiltrating myeloid populations, including cDCs (CD11c^+^MHC‐II^+^), monocytic myeloid cells/M‐MDSCs (Ly6C^+^Ly6G^−^), granulocytic myeloid cells/PMN‐MDSCs (Ly6G^+^Ly6C^−^), M1‐like macrophages, and M2‐like macrophages within the CD11b^+^ myeloid compartment. *n* = 5 per group. One‐way ANOVA. Scale bars are shown in the corresponding panels. Data are presented as mean ± SD. *ns, not significant*; ^*^ *p* < 0.05, ^**^ *p* < 0.01, ^***^ *p* < 0.001, ^****^ *p* < 0.0001.

This candidate pathway was then validated at the protein and transcript levels. IF reduced ATF4, CHOP, and ERO1A protein expression in tumor tissues (Figure 6B). Quantitative PCR further confirmed the reduction in Ero1a mRNA expression after IF (Figure S7C). Consistent with the proposed PERK dependency, PERK KD reduced ERO1A expression, and taurine supplementation failed to restore ERO1A after PERK depletion (Figure S7J,K). GRP78 knockdown weakened the ability of taurine to preserve PERK and downstream signaling under stress, supporting a GRP78‐dependent mechanism for taurine‐mediated PERK maintenance (Figure S8A,B).

The functional role of ERO1A was next examined in vivo. ERO1A overexpression accelerated tumor growth and increased final tumor weight, whereas ERO1A knockdown suppressed tumor growth and reduced tumor burden (Figure 6C–H). Immunofluorescence analysis showed that ERO1A overexpression increased Ki‐67 staining and reduced CD^3+^ CD^8+^ T‐cell infiltration. ERO1A knockdown produced the opposite phenotype, with reduced tumor‐cell proliferation and increased T‐cell infiltration (Figure 6I). Immune profiling further showed that ERO1A overexpression reduced CD^8+^ T‐cell infiltration and activation, whereas ERO1A KD enhanced CD^8^^+^ T‐cell effector features and shifted tumor‐infiltrating myeloid cells toward a more antigen‐presenting and less suppressive state (Figure 6J; Figure S9A). Consistently, ERO1A KD shifted tumor‐infiltrating myeloid cells toward a more antigen‐presenting and less suppressive state, whereas ERO1A overexpression produced the opposite pattern (Figure 6K; Figure S9B).

Because ERO1A is a downstream effector of PERK signaling, an in vivo rescue experiment was performed to determine whether reduced ERO1A contributes to the antitumor phenotype caused by PERK depletion. ERO1A overexpression did not significantly alter endpoint tumor weight in PERK‐WT tumors. In contrast, PERK knockdown markedly inhibited tumor growth, whereas ERO1A overexpression partially restored tumor volume and weight in PERK‐deficient tumors to levels comparable to those of PERK‐WT controls (Figure S10A–C).

Immunofluorescence staining showed increased intratumoral CD^3^ and CD^8^ signals in PERK‐KD tumors, accompanied by stronger CD86 and weaker CD206 staining. These changes were attenuated following ERO1A overexpression (Figure S10D). Flow cytometry further demonstrated that PERK knockdown increased the intratumoral proportion of CD^3^^+^CD^8^^+^ T cells and markedly enhanced the frequencies of GZMB^+^ and IFN‐γ^+^ cells within the CD^8^^+^ T‐cell population. ERO1A overexpression significantly reduced these effects in PERK‐KD tumors (Figure S10E). Thus, restoration of ERO1A substantially counteracted both the tumor suppression and CD^8^^+^ T‐cell activation associated with PERK depletion, supporting ERO1A as a functional downstream mediator of PERK in CRC.

Human tissue analyses further supported the clinical relevance of this pathway. High PERK expression was associated with reduced CD^8^^+^ T‐cell infiltration and poorer overall survival (Figure S11A–C). Similarly, high ERO1A expression correlated negatively with CD^8^^+^ T‐cell infiltration and showed a trend toward poorer survival (Figure S11D–F).

### 2.7 Taurine Restriction Supports Tumor Control in Combination With Anti‐PD‐1 Therapy

The therapeutic effect of combining taurine restriction with PD‐1 blockade was evaluated in syngeneic CRC models. In MC38 tumors, IF combined with anti‐PD‐1 therapy produced a greater suppression trend of tumor growth and final tumor weight than either IF or anti‐PD‐1 treatment alone (Figure 7A–C). A similar combined effect was observed in CT26 tumors, where IF plus anti‐PD‐1 further reduced tumor progression compared with control or monotherapy groups (Figure 7D–F). Pharmacological restriction of taurine uptake with GES also strengthened the antitumor effect of anti‐PD‐1 therapy in CT26 tumors (Figure 7G–I).

**FIGURE 7:**
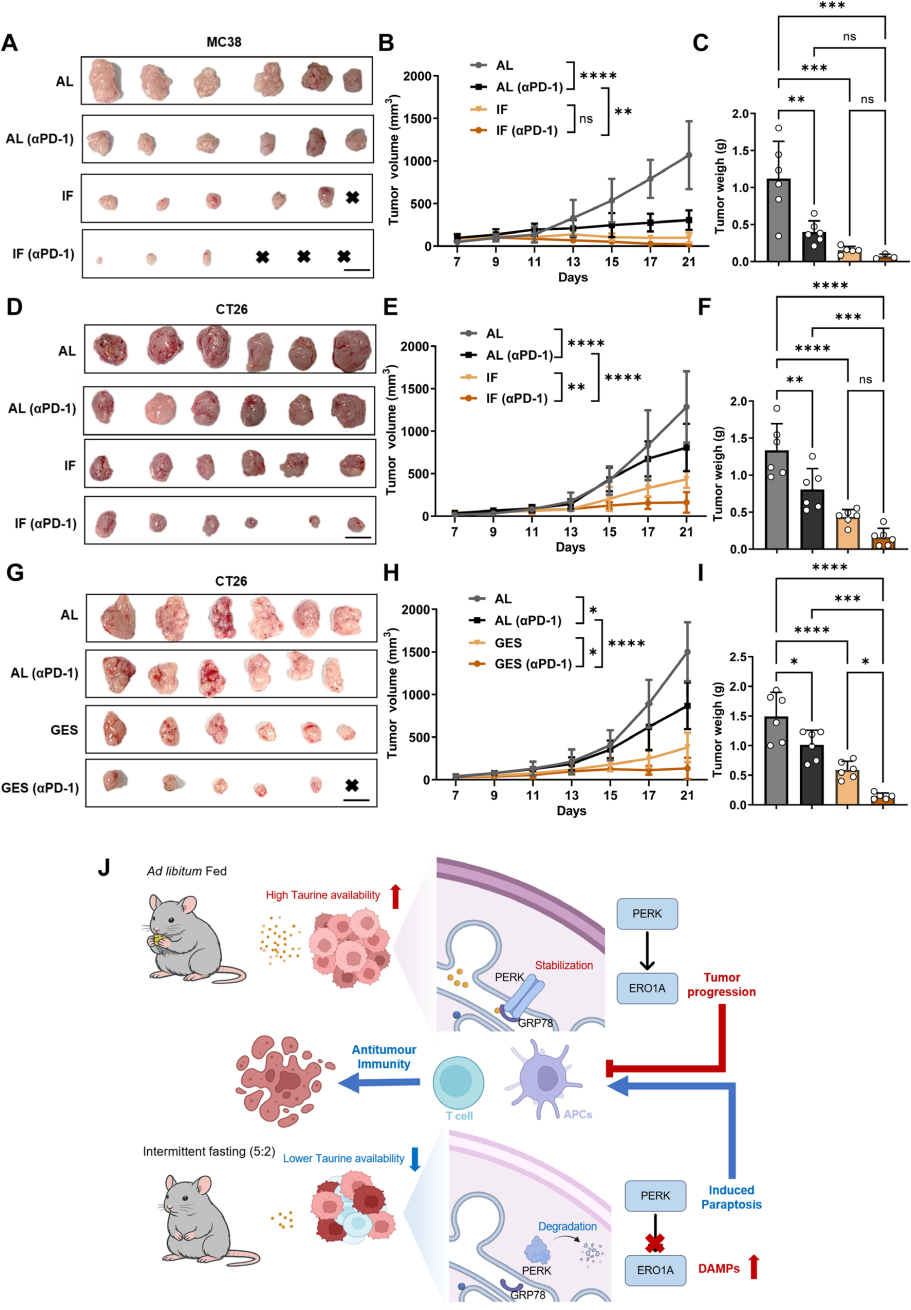
Taurine restriction combines with PD‐1 blockade to improve tumor control. (A–C) Representative tumor images (A), tumor growth curves (B), and tumor weight quantification (C) from MC38 tumor‐bearing mice treated with AL plus isotype IgG, anti‐PD‐1, IF plus isotype IgG, or IF plus anti‐PD‐1. *n* = 3–6 per group. Two‐way ANOVA for tumor growth curves; one‐way ANOVA for tumor weight. (D‐F) Representative tumor images (D), tumor growth curves (E), and tumor weight quantification (F) from CT26 tumor‐bearing mice treated with AL plus isotype IgG, anti‐PD‐1, IF plus isotype IgG, or IF plus anti‐PD‐1. *n* = 6 per group. Two‐way ANOVA for tumor growth curves; one‐way ANOVA for tumor weight. (G–I) Representative tumor images (G), tumor growth curves (H), and tumor weight quantification (I) from CT26 tumor‐bearing mice treated with AL plus isotype IgG, anti‐PD‐1, GES plus isotype IgG, or GES plus anti‐PD‐1. *n* = 5–6 per group. Two‐way ANOVA for tumor growth curves; one‐way ANOVA for tumor weight. (J) Proposed model. IF restricts systemic and intratumoral taurine availability, disrupting taurine‐GRP78‐ associate PERK stabilization. PERK loss suppresses the ATF4‐CHOP‐ERO1A stress‐adaptation axis and induces paraptosis‐like immunogenic stress, thereby promoting CD8^+^ T‐cell‐mediated antitumor immunity and providing a mechanistic basis for combining taurine restriction with PD‐1 blockade. Scale bars are shown in the corresponding panels. Data are presented as mean ± SD. *ns, not significant*; ^*^ *p* < 0.05, ^**^ *p* < 0.01, ^***^ *p* < 0.001, ^****^ *p* < 0.0001.

The proposed model is that IF lowers systemic and intratumoral taurine availability, thereby disrupting taurine‐GRP78‐associate PERK stabilization. PERK loss weakens the downstream axis and induces paraptosis‐like immunogenic stress. This tumor‐intrinsic stress program promotes CD8^+^ T‐cell‐mediated antitumor immunity and provides a mechanistic basis for combining taurine restriction with anti‐PD‐1 therapy (Figure 7J).

## 3 Discussion

Fasting‐related interventions are understood as biologically active modulators of tumor behavior rather than as simple reductions in caloric intake [20]. The fasting‐mimicking diet has been demonstrated to enhance antitumor immunity by reducing intratumoral IgA‐producing B cells [21]. Recent work has linked fasting‐associated microbiota remodeling to enrichment of Bifidobacterium pseudolongum, increased L‐arginine levels, and promoted expansion of memory CD8^+^ T cells [3]. These studies indicate that fasting engages multiple metabolic and immune programs that contribute to tumor control in CRC.

IF induces broad systemic metabolic and endocrine remodeling [22]. Previous studies have demonstrated that high circulating taurine is associated with an increased risk of CRC, whereas elevated taurine levels correlate with more advanced disease [23]. In our serum metabolomic analysis, taurine was among the metabolites most substantially reduced by IF, indicating that IF decreases taurine availability to tumor cells. Consistently, taurine abundance was also decreased in TIF and tumor tissues, indicating that systemic taurine restriction extends to the local tumor compartment [11]. Taurine utilization has been implicated in tumor survival, stem‐like properties, and multidrug resistance in CRC [24, 25]. Taurine derived from the tumor microenvironment can also suppress ferroptosis in malignant cells, while low‐dose taurine supplementation has been reported to promote tumor growth in immunocompetent lung cancer models [26, 27]. Conversely, taurine availability can support CD8^+^ T‐cell survival and effector function [11]. SLC6A6‐high tumor cells competitively consumed taurine, resulting specifically in taurine deficiency within CD8^+^ T cells and ER‐stress‐associated exhaustion [11]. However, SLC6A6 expression reflects cellular taurine‐uptake capacity rather than extracellular taurine abundance and therefore does not necessarily predict TIF taurine concentration [15]. TIF represents the extracellular nutrient pool available to tumor‐resident cells, and its metabolite composition is influenced by multiple local and systemic determinants [28, 29]. This T‐cell‐intrinsic taurine‐deficiency phenotype is biologically distinct from the systemic and bulk intratumoral reduction in taurine availability. Pharmacological inhibition of taurine uptake similarly suppressed tumor growth, while the reduction in taurine during IF was accompanied by enhanced rather than impaired CD^8^^+^ T‐cell activity. These observations suggest that IF‐associated taurine restriction acts, through impaired tumor‐cell stress adaptation, with increased tumor immunogenicity subsequently contributing to the CD^8^^+^ T‐cell response.

Previous research has demonstrated that taurine restriction exacerbated ER stress and promoted PERK‐associated cell death [30]. However, the molecular mediator through which taurine influences stress adaptation in CRC remains unclear. In this study, GRP78 was identified and validated as a taurine‐associated protein in CRC. Functional analyses further supported GRP78 as a key mediator of taurine‐dependent PERK maintenance. GRP78 is a central chaperone in the ER stress response [31]. Under basal conditions, it binds to ER stress sensors and keeps them inactive [32]. Nutrient deprivation, hypoxia, inflammatory cues, and the accumulation of misfolded proteins disrupt ER homeostasis and release GRP78 from these sensors, thereby activating the unfolded protein response [33]. PERK has been linked to survival under glucose restriction in cancer cells, indicating that this axis functions as a pro‐survival buffer under hostile metabolic conditions [34, 35]. PERK ablation in ER‐stressed malignant cells has been shown to induce paraptosis with immunogenic consequences, suggesting that reduced PERK signaling may redirect nutrient stress away from adaptive buffering and toward paraptosis [36]. Our study showed that under nutrient stress, reduced taurine availability was associated with lower PERK protein abundance.

Paraptosis is a non‐apoptotic death program characterized by extensive cytoplasmic vacuolization and swelling of the endoplasmic reticulum and/or mitochondria [37]. Paraptosis has emerged as a therapeutically relevant form of regulated cell death in cancer [38, 39]. Beyond its direct cytotoxic effects, paraptotic cell death may also enhance antitumor immunity and improve responses to immunotherapy [40]. Experimental evidence suggests that paraptosis can alter the tumor microenvironment, promote immune activation, and elicit immunogenic cell death, thereby contributing to improved therapeutic outcomes [41]. In our study, reduced taurine availability under IF was associated with lower PERK stability under nutrient stress and with paraptosis accompanied by increased immunogenic signal, enhanced antigen‐presenting cell accumulation, and CD^8^^+^ T‐cell effector activity. In our study, reduced taurine availability under IF was associated with lower PERK stability under nutrient stress and with paraptosis accompanied by increased immunogenic signals, enhanced antigen‐presenting cell accumulation, and CD^8^^+^ T‐cell effector activity. These tumor‐immunogenic changes may contribute to the improved tumor control during anti‐PD‐1 treatment in our models. The apparently divergent effects of taurine manipulation on PD‐1 blockade may reflect distinct cellular mechanisms, with taurine supplementation supporting T‐cell fitness in some contexts, whereas restriction of tumor‐cell taurine availability may increase tumor immunogenicity and thereby support tumor control during checkpoint blockade in others [42, 43].

This study has several limitations. First, the precise structural basis by which taurine‐associated GRP78 modulates GRP78‐PERK association remains to be further defined. Molecular docking and AI‐assisted structure prediction are also unable to yield a reliable high‐confidence model, particularly for the local taurine‐associated pose and GRP78‐PERK interface. Second, because IF engages multiple metabolic and immune programs, future studies directly comparing IF responses in PERK‐proficient and PERK‐deficient tumors will be needed to define the quantitative contribution of PERK to the overall fasting response. Third, in the clinical analysis, taurine abundance could not be measured in the same cohort specimens used for SLC_6_A_6_, CD^8^^+^ T‐cell infiltration, and survival analyses because paired fresh‐frozen tissues or blood samples were unavailable. Future studies with paired metabolomic, transporter‐expression, and immune‐profiling data from the same tumors will be needed to further define the quantitative relationship among taurine abundance, SLC6A6 expression, and antitumor immune infiltration.

## 4 Conclusion

This study identified a tumor‐intrinsic mechanism by which IF enhances antitumor immunity in CRC. Our data support PERK attenuation as a functional downstream component of fasting‐associated taurine restriction within the broader metabolic response to IF. Reduced taurine availability during IF was associated with lower PERK stability, paraptosis‐like cell death with immunogenic features, and improved tumor control during PD‐1 blockade in CRC models.

## 5 Methods

### 5.1 Ethical Statement

The use of patient‐derived specimens was approved by the Ethics Committee of Zhongshan Hospital, Fudan University (B2021‐777R), whereas the tissue microarray study was approved by the Ethics Committee of Shanghai Outdo Biotech Co., Ltd. (SHYJS‐CP‐1901003). All procedures complied with the principles of the Declaration of Helsinki.

For cohort1, patients were included if they were newly diagnosed with primary colorectal adenocarcinoma, had not received neoadjuvant chemotherapy or radiotherapy before surgery, and underwent surgical resection. Availability of evaluable tumor tissue and complete clinicopathological and follow‐up information was also required. Patients were excluded if they had non‐adenocarcinoma histology, had received any neoadjuvant treatment, or had insufficient tissue or incomplete follow‐up data for the planned analyses.

For cohort 2, fresh‐frozen specimens were obtained during endoscopic examination from 23 patients with colorectal cancer who had not received prior treatment. For each patient, tumor tissue, matched adenomatous tissue, and matched non‐neoplastic colonic mucosa were collected. Non‐neoplastic samples were obtained from sites located at least 10 cm from the tumor.

Peripheral blood was collected from healthy participants. Eligibility criteria included a colonoscopic examination showing no colorectal neoplasia or polyps, no history of malignant disease, and no infectious disease within the preceding 4 weeks.

### 5.2 Mouse

#### 5.2.1 Treatments and Subcutaneous Cancer Mouse Model

Male C57BL/6JGpt (N000013), BALB/cJGpt (N000020), and Apc^Min/+^ mice (T001457), aged 6–8 weeks, were obtained from GemPharmatech Co., Ltd. (China). All animal procedures were approved by the Ethics Committee of Zhongshan Hospital, Fudan University (2025‐060).

For subcutaneous tumor implantation, CT26 cells were inoculated into BALB/c mice, whereas MC38 cells were implanted into C57BL/6 mice. Cells were collected, resuspended in PBS, and injected subcutaneously into the flank at a dose of 2 × 10^5 cells per mouse. Tumor volume was calculated using the standard formula: volume = length × width^2^ / 2.

To assess systemic antitumor immune effects, we established a sequential bilateral flank tumor model in which PERK WT or PERK KD MC38 cells were first implanted into one flank, followed 7 days later by inoculation of tumor cells into the contralateral flank.

Ad libitum (AL) group had no restricted access to food and water. The intermittent fasting (IF) regimen consisted of two 24 h fasting periods on nonconsecutive days each week, during which water remained freely available.

For taurine‐supplementation experiments, taurine was administered by oral gavage at 100 mg/kg once daily. Treatment was initiated on day 7 after tumor implantation and continued until tumor collection.

For taurine‐uptake inhibition, guanidinoethyl sulfonate (GES) was dissolved in drinking water at the concentration of 1% (w/v), and mice were allowed free access to the GES‐containing water throughout the treatment period. GES treatment was initiated on day 7 after tumor implantation and continued until the experimental endpoint.

For CD8^+^ T‐cell depletion, mice received intraperitoneal injections of anti‐mouse CD8α antibody (200 µg per mouse; clone YTS 169.4, Cat. No. T9901A‐629; TargetMol, USA) or the corresponding IgG2a κ isotype control (Cat. No. T78337; TargetMol, USA) twice weekly. For PD‐1 blockade, anti‐mouse PD‐1 antibody (200 µg per mouse; clone RMP1‐14, Cat. No. T78269; TargetMol, USA) or the matched isotype control was administered intraperitoneally on the same schedule.

#### 5.2.2 Randomization

Formal randomization was not used to allocate experimental units to control and treatment groups. Animals were assigned to experimental groups after confirmation of tumor establishment, with efforts made to balance baseline tumor size and body weight across groups. Potential confounders were minimized by using mice of similar age, maintaining identical housing conditions, and handling animals from different groups in parallel whenever possible. Tumor measurements, tissue collection, and downstream assays were performed using the same procedures across groups. No additional measures were taken to control for cage location or treatment order.

#### 5.2.3 Sample Size

Sample size was estimated using the resource equation approach. For a one‐way ANOVA design with k groups and n animals per group, the error degrees of freedom were defined as E = N − k = k(*n* − 1). To obtain the minimum sample size, E was set at 10, yielding *n* = 10/k + 1, which was rounded up to the nearest whole number.

### 5.3 Cell Culture

Human DLD‐1 colorectal carcinoma cells (TCHu134) and murine MC38 (SCSP‐5431) and CT26.WT (TCM37) colorectal carcinoma cells were obtained from the CAS Center for Excellence in Molecular Cell Science (China). Cells were cultured in Dulbecco's modified Eagle's medium (DMEM; MA0212, MeilunBio, China) containing 10% fetal bovine serum (FBS) (PWL217, MeilunBio, China) at 37°C in a humidified atmosphere with 5% CO_2_.

Unless otherwise indicated, tumor cells were exposed for 24 h to short‐term starvation medium consisting of glucose‐free DMEM (MA0581, MeilunBio, China) with 0.5 g/L glucose (BGC001, MeilunBio, China) and 1% FBS. Where indicated, the medium was supplemented with taurine (200 µm; T0022, TargetMol, USA), Z‐VAD‐FMK (20 µm; T7020), necrostatin‐1 (20 µm; T1847), ferrostatin‐1 (10 µm; T6500), dapansutrile (10 µm; T15052), chloroquine (10 µm; T8689), or AZ9482 (10 µm; T22264).

Human peripheral blood mononuclear cells (PBMCs) were isolated from healthy‐donor blood by Ficoll‐Paque Premium density‐gradient centrifugation (Cat. No.17544202, Cytiva, USA). Freshly isolated PBMCs were activated using the ImmunoCult Human CD3/CD28 T Cell Activator (100‐0784, STEMCELL Technologies, Canada) and maintained in ImmunoCult‐XF medium (100‐0956, STEMCELL Technologies, Canada) supplemented with 10 ng/mL recombinant human IL‐2 (MB5951, MeilunBio, China).

### 5.4 Flow Cytometry

Freshly excised tumors were mechanically minced and enzymatically dissociated with Liberase and DNase I (Roche, USA). Erythrocytes were subsequently removed using ammonium–chloride–potassium lysis buffer. The resulting cell suspensions were passed through sterile 100‐µm strainers to obtain single cells for flow‐cytometric analysis.

All fluorophore‐conjugated antibodies and probes are detailed in Table S3. Viable cells were identified using viability dye (Cat. No. 65‐0865‐14; Thermo Fisher Scientific, USA) before antibody labeling. For surface‐marker detection, cells were incubated with the indicated antibodies with the Fc receptor‐blocking reagent (Cat. No. 553142; BD Biosciences, USA). Intracellular and intranuclear staining was performed using the fixation and permeabilization reagent (Cat. No. 00‐5523‐00; Thermo Fisher Scientific).

For analysis of IFN‐γ, TNF‐α, and granzyme B in T cells, the single‐cell suspensions were stimulated with cell stimulation cocktail (Cat. No. 00–4975; Thermo Fisher Scientific) before surface and intracellular staining. Ki‐67 staining was performed after surface labeling, fixation, and permeabilization.

Cell death was assessed using apoptosis detection kit (Cat. No. MA0429; MeilunBio, China). Endoplasmic reticulum morphology was evaluated with ER‐Tracker Red (Cat. No. C1041S; Beyotime, China), and DNA synthesis was measured using an Alexa Fluor 555 EdU proliferation assay kit (Cat. No. MA0425‐1; MeilunBio). All these assays were performed according to the manufacturers’ instructions.

### 5.5 Tumor Interstitial Fluid (TIF) Collection

TIF was isolated from subcutaneous mouse tumors. Freshly excised tumors were rinsed briefly with PBS, minced, and transferred onto 70‐µm cell strainers positioned over collection tubes. Samples were centrifuged at 400 × g, and the recovered fluid was filtered with 0.22‐µm membranes.

### 5.6 Taurine Measurement

Taurine levels in serum, tumor interstitial fluid, mouse tumor tissues, human tissue samples, and tumor cell lysates were measured using the taurine assay kit (Cat. No. abs580222, Absin, China). For tissue and cell samples, taurine levels were normalized to protein concentration. For serum and TIF samples, taurine levels were reported as concentrations in the collected fluid.

### 5.7 Detection of Immunogenic Cell Death Markers

ExoCRT was detected via flow cytometry and cell immunofluorescent staining via surface labelling with CoraLite Plus 750‐conjugated calreticulin recombinant monoclonal antibody (Cat. No.CL750‐80017, Proteintech, China). HMGB1 and IFN‐β were detected in TIF with the mouse HMGB1 ELISA kit (Cat. No.EEL102, Thermo Fisher Scientific Inc., USA) or mouse IFN‐β ELISA kit (Cat. No.DIFNB0, R&D Systems, USA). HMGB1 and IFN‐β release were detected in the medium of human cell lines via the human HMGB1 ELISA kit (Cat. No.EEL047, Thermo Fisher Scientific Inc., USA) or the IFN‐β ELISA kit (Cat. No.42400‐1, R&D Systems, USA).

### 5.8 Antitumor Effect Assay Using the Luciferase Reporter

PBMC‐mediated tumor‐cell killing was assessed using luciferase‐expressing DLD‐1 cells. PBMCs and DLD‐1 cells were directly co‐cultured at an effector‐to‐target ratio of 5:1 for 48 h. Firefly luciferase activity was measured using a reporter assay kit (Cat. No. RG005; Beyotime, China). Luminescence values were normalized to those of DLD‐1 cells cultured without PBMCs and expressed as normalized relative light units (RLU). Lower normalized RLU values indicated fewer viable tumor cells and greater PBMC‐mediated cytotoxicity.

### 5.9 Cell Proliferation Assay

Cells receiving the indicated treatments were plated in 96‐well plates. At the specified time points, Cell Counting Kit‐8 reagent (Cat. No. C0037; Beyotime, China) was added to each well, followed by incubation at 37°C. Absorbance at 450 nm was measured.

### 5.10 Multiplex Immunohistochemistry

Multiplex immunofluorescence staining of human tissues was performed using the Opal 7‐color manual IHC kit (Cat. No. NEL811001KT; PerkinElmer, USA). Following antigen retrieval, sections were sequentially incubated with antibodies against SLC6A6 (ab236898; Abcam), CD8 (ab217344; Abcam), PERK/EIF2AK3 (24390‐1‐AP; Proteintech), and ERO1A (12007‐1‐AP; Proteintech), with heat‐mediated antibody stripping between staining cycles. Signals were detected using the corresponding Opal fluorophores, and nuclei were counterstained with spectral DAPI.

The entire evaluable tumor area was analyzed. Marker expression was quantified as the percentage of positive cells and treated as a continuous variable in correlation analyses. For survival‐based stratification, ROC‐derived Youden‐index cutoffs were used: SLC6A6, 17.35%; PERK, 62.57%; and ERO1A, 59.31%.

Mouse FFPE tumor sections were stained sequentially for CD3, CD8, CD206, CD86 and Ki67 using horseradish peroxidase‐mediated tyramide signal amplification, with heat‐induced stripping between cycles and DAPI counterstaining. Five randomly selected non‐overlapping regions were quantified per section, and their mean value was used as one biological replicate.

### 5.11 Generation of Genetically Modified Cell Lines

Genetically modified DLD1 and MC38 cells were generated using lentiviral vectors. For human DLD1 cells, EIF2AK3/PERK knockdown was generated using a GV493 shRNA lentiviral vector targeting human EIF2AK3. The EIF2AK3‐targeting sequence used in this study was GCACACAGATTACAGTCAGAT. HSPA5/GRP78 knockdown in DLD1 cells was generated using a GV493 shRNA lentiviral vector targeting human HSPA5. The HSPA5‐targeting sequence was GAAATCGAAAGGATGGTTAAT.

For mouse MC38 cells, Eif2ak3/Perk knockdown and Ero1a knockdown were generated using GV112 shRNA lentiviral vectors targeting mouse Eif2ak3 and Ero1a, respectively. The mouse Eif2ak3‐targeting sequence was GCCACTTTGAACTTCGGTATA, and the mouse Ero1a‐targeting sequence was GTCCTAAGCCAAACGTTTATA. Ero1a overexpressing MC38 cells were generated using the GV308 lentiviral vector carrying the full‐length mouse Ero1a coding sequence.

Control cells were transduced with the corresponding non‐targeting shRNA or empty‐vector control. After lentiviral transduction, stable cells were selected with puromycin, and knockdown and overexpression efficiency were confirmed by qPCR and western blotting before subsequent experiments.

### 5.12 Quantitative RT‐PCR

Total RNA was extracted from tumor tissues or cultured cells using TRIzol reagent (Cat. No. 15596026CN; Thermo Fisher Scientific, USA) and reverse‐transcribed with the PrimeScript RT Reagent Kit (Cat. No. RR047B; Takara, Japan). Quantitative PCR was performed using TB Green Fast qPCR Mix (Cat. No. RR430B; Takara) on an Applied Biosystems 7900HT system. Primer sequences are provided in Table S3.

### 5.13 Western Blot

Cell and tissue lysates were prepared using M‐PER reagent (Cat. No. 78501; Thermo Fisher Scientific, USA), and protein concentrations were measured. Protein samples were resolved by 10% SDS‐PAGE, transferred to PVDF membranes (Cat. No. SEQ85R; Merck Millipore, USA), and probed with primary antibodies followed by secondary antibodies. Signals were detected using the ECL substrate (Cat. No. MA0186‐2; MeilunBio, China). Unless otherwise stated, immunoblotting was performed in three independent biological replicates.

### 5.14 Drug Affinity Responsive Target Stability (DARTS) Assay

For DARTS analysis, DLD1 cells were lysed in lysis buffer. Protein concentrations were measured, and equal amounts of lysates were incubated with taurine (0–100 µm) at 37°C for 1 h. Proteinase K (10 µg/mL) was then added, followed by further incubation to facilitate proteolytic digestion. LC‐MS/MS was subsequently used to analyze samples and identify potential target proteins of taurine. Independent experiments were performed using the same protocol, and candidate targets were validated by western blotting.

### 5.15 Biotinylated Taurine Pulldown Assay

For biotinylated taurine pulldown assays, cells were lysed in the lysis buffer with protease inhibitors. Cell lysates were incubated with biotinylated taurine at 4°C with rotation. For competition assays, excess unlabeled taurine was added before incubation with biotinylated taurine. Biotinylated taurine‐associated protein complexes were captured using streptavidin magnetic beads, followed by extensive washing to remove nonspecific binding. The bound proteins were eluted and analyzed with anti‐GRP78 antibody. Input lysates were analyzed in parallel.

### 5.16 Cycloheximide Chase Assay and Trafficking Inhibitor Assay

To evaluate PERK protein stability, cells were subjected to short‐term starvation (STS) with or without taurine. Cycloheximide (CHX, 100 µg/mL) was then added to block de novo protein synthesis.

To explore the pathway involved in PERK protein loss under nutrient stress, cells were exposed to STS in the presence or absence of inhibitors targeting major protein quality‐control or trafficking pathways. MG132 was used as a proteasome inhibitor; chloroquine and 3‐methyladenine were used as inhibitors of the autophagy‐related pathway; and brefeldin A was used as an ER‐Golgi trafficking inhibitor. Cells were harvested after 24 h for western blot analysis of PERK protein abundance.

### 5.17 Co‐Immunoprecipitation

For analysis of the GRP78‐PERK interaction, co‐immunoprecipitation was performed using the IP/Co‐IP kit (Cat. No. 88804; Thermo Fisher Scientific, USA) according to the manufacturer's instructions. Cell lysates were incubated with anti‐GRP78 antibody or control IgG, and immune complexes were captured using the magnetic beads supplied with the kit. After washing and elution, the precipitated proteins were analyzed by immunoblotting for PERK and GRP78. Corresponding input lysates were examined in parallel.

### 5.18 PERK Ubiquitination Assay

PERK ubiquitination was assessed in cells subjected to control, STS, or STS plus taurine treatment. MG132 was added 24 h before collection. Cells were lysed in immunoprecipitation buffer. Equal amounts of lysate were immunoprecipitated with anti‐PERK antibody and protein A/G magnetic beads. After washing and elution, ubiquitinated PERK was detected with the anti‐ubiquitin antibody. Total PERK in the immunoprecipitates and corresponding input lysates were analyzed in parallel.

### 5.19 Serum Metabolomic Profiling

Serum was obtained from AL‐ and IF‐treated Apc^min/+^ mice by centrifugation of whole blood. Metabolites were extracted with prechilled methanol/acetonitrile, followed by protein precipitation and clarification by centrifugation. Untargeted metabolomic analysis and chromatographic separation was performed. Data were collected in positive‐ and negative‐ion electrospray modes using data‐dependent acquisition. Serum metabolomic profiling was conducted by OEBiotech (Shanghai, China).

### 5.20 Bulk RNA Sequencing

Bulk RNA sequencing was performed using tumor tissues from AL‐ and IF‐fed Apc^min/+^ mice, with 3 biological replicates per group. Total RNA was extracted and used for library preparation. Libraries were sequenced with 150‐bp paired‐end reads.

Raw reads were filtered with fastp and aligned to the GRCm39/mm39 mouse genome using HISAT2. Gene counts were generated with HTSeq‐count, and differential expression was analyzed using DESeq2. Gene‐set enrichment analysis (GSEA) was performed using ranked gene lists to identify coordinated pathway‐level changes after IF. Predefined gene sets related to ER stress, CHOP‐associated stress response, protein folding, translation, glycolysis, adaptive immune response, CD8^+^ T‐cell response, and antigen processing were analyzed. Bulk RNA sequencing was conducted by OEBiotech (Shanghai, China).

### 5.21 Quantification and Statistical Analysis

Statistical analyses were performed using GraphPad Prism 7 (GraphPad Software, San Diego, CA, USA). Data are presented as mean ± SD unless otherwise indicated. Comparisons between two groups were performed using a two‐tailed unpaired *t*‐test or paired *t*‐test, as appropriate. Comparisons among multiple groups were performed using ordinary one‐way ANOVA, followed by appropriate multiple‐comparisons tests. For longitudinal tumor growth curves and body weight, repeated measurements from the same animals were analyzed using two‐way repeated‐measures ANOVA. Survival was analyzed by the Kaplan–Meier method with the log‐rank test. Correlations were assessed using Pearson's correlation analysis. A two‐sided *p* value < 0.05 was considered statistically significant. Statistical details for each analysis are provided in the corresponding figure legends.

## Author Contributions

**Baohui Song**: conceptualization; investigation; writing – original draft; writing – review editing; visualization; validation; methodology; software; formal analysis; data curation; supervision; resources. **Sikei Kam**: conceptualization; investigation; writing – original draft; writing – review editing; visualization; validation; methodology; software; formal analysis; data curation; supervision; resources. **Jingyi Liu**: data curation; supervision; formal analysis; software; resources; writing – original draft; writing – review editing; visualization; methodology; validation; conceptualization; investigation. **Rui Lv**: investigation; writing – review editing; methodology; validation; formal analysis; data curation. **Xucheng Huo**: methodology; data curation; resources. **Zhanghan Chen**: resources; writing – review editing; methodology; validation. **Yuelun Dong**: methodology. **Shilun Cai**: methodology; conceptualization; project administration; visualization. **Bing Li**: project administration; conceptualization. **Ruobing Ren**: conceptualization; investigation; funding acquisition; writing – review editing; methodology; project administration. **Yunshi Zhong**: project administration; supervision; resources; writing – review editing; conceptualization; writing – original draft; funding acquisition. **Mingyan Cai**: funding acquisition; writing – original draft; conceptualization; methodology; writing – review editing; project administration; supervision.

## Funding

This work was supported by Shanghai Municipal Health Commission Specialized Clinical Research Project (Grant No. 202340178 and 202540081), National Natural Science Foundation of China (Grant No. 62635004 and 82273025), Clinical Research Special Project of Zhongshan Hospital, Fudan University (Grant No. ZSLCYJ202350), and the Shanghai Innovative Medical Devices and Pharmaceuticals Application Demonstration Project (Grant No. 25SF1906800).

## Conflicts of Interest

The authors declare no conflicts of interest.

## Supporting information




**Supporting File**: advs78107‐sup‐0001‐SuppMat.pdf.

## Data Availability

The RNA‐seq and serum metabolomics datasets generated in this study are available in the OMIX repository under accession numbers OMIX017225 and OMIX017226, respectively.
