# Supplementary material for: Intermittent Fasting Reduces Taurine Availability and Promotes Antitumor Immunity Through PERK Attenuation‐Associated Paraptosis‐Like Cell Death in Colorectal Cancer
Source: Adv Sci (Weinh). 2026 Oct 6:e78107. Online ahead of print. doi: 10.1002/advs.78107 (PMC13643236; doi:10.1002/advs.78107)
Supplement: Supplementary file 1 [file ADVS-9999-e78107-s001.pdf]

Supporting Information

Intermittent fasting reduces taurine availability and promotes antitumor immunity through PERK attenuation-associated paraptosis-like cell death in colorectal cancer

Baohui Song, Sikei Kam, Jingyi Liu, Rui Lv, Xucheng Huo, Zhanghan Chen, Yuelun Dong, Shilun Cai, Bing Li, Ruobing Ren, Yunshi Zhong* , Mingyan Cai**

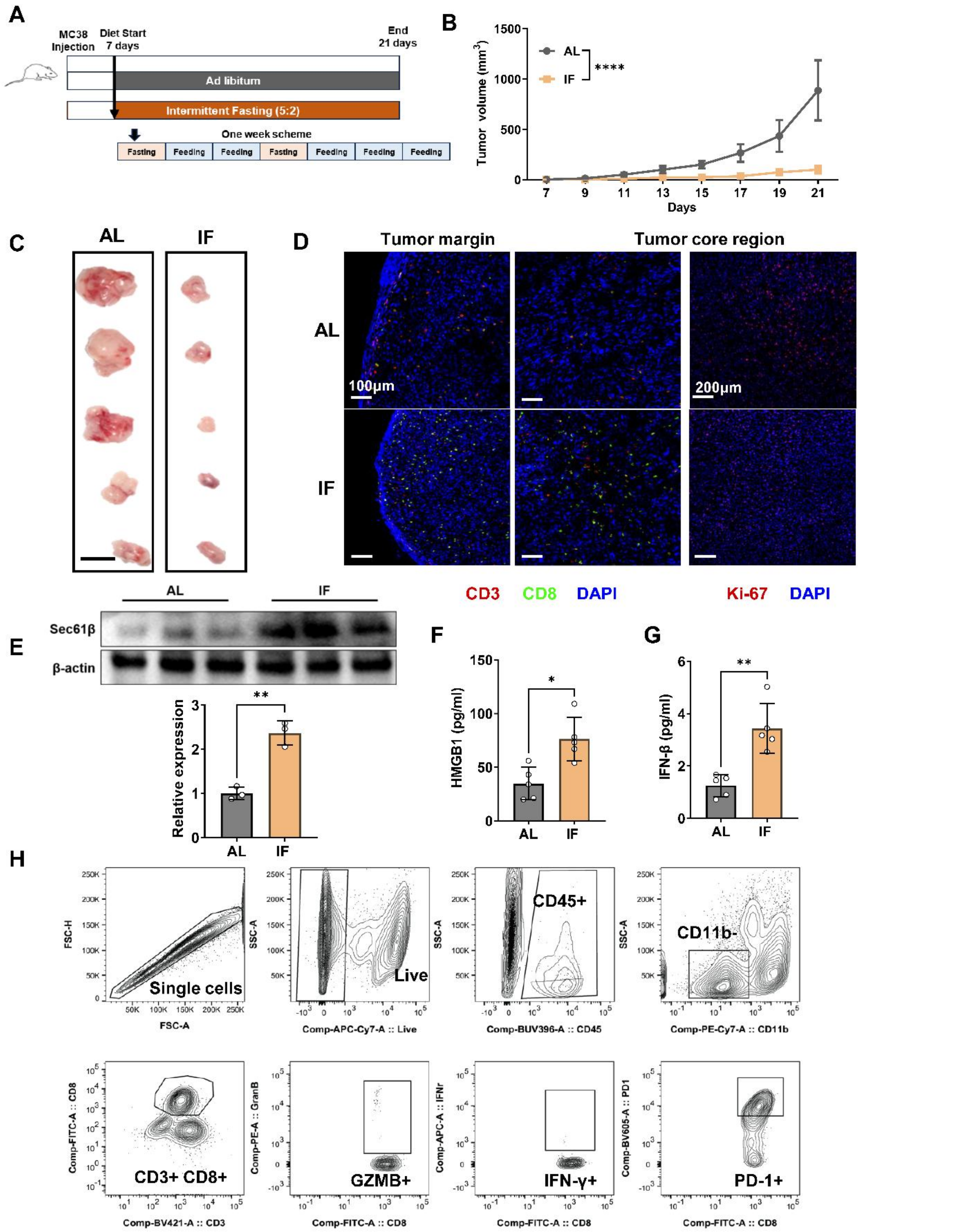

Figure S1. Intermittent fasting suppressed MC38 tumor growth and induces immunogenic stress.
(A) Schematic of the 5:2 intermittent fasting (IF) regimen in the MC38 syngeneic tumor model. Mice were maintained under ad libitum feeding (AL) or IF until endpoint analysis.
(B, C) Tumor growth curves (B) and representative tumor images (C) from AL- and IF-fed MC38 tumor-bearing mice. n = 5 mice per group. Two-way ANOVA for tumor growth curves.

(D) Representative immunofluorescence images showing CD3, CD8, and Ki-67 staining in tumor margin and tumor core regions from AL- and IF-fed tumors.

(E) Western blot analysis and quantification of SEC61β expression in AL- and IF-treated tumors. n = 3 biological replicates per group. Unpaired t-test.

(F, G) Tumor interstitial fluid HMGB1 (F) and IFN-β (G) levels in AL- and IF-fed tumor-bearing mice. n = 5 mice per group. Unpaired t-test.

(H) Gating strategy for tumor-infiltrating immune cells, including single cells, live cells, $CD45^+$ cells, $CD11b^-$ lymphoid cells, $CD3^+CD8^+$ T cells, and $GZMB^+$, IFN-γ^+, and PD-1^+ $CD8^+$ T-cell subsets.

Scale bars are shown in the corresponding panels. Data are presented as mean ± SD.

$^*P < 0.05$, $^{**}P < 0.01$, $^{****}P < 0.0001$.

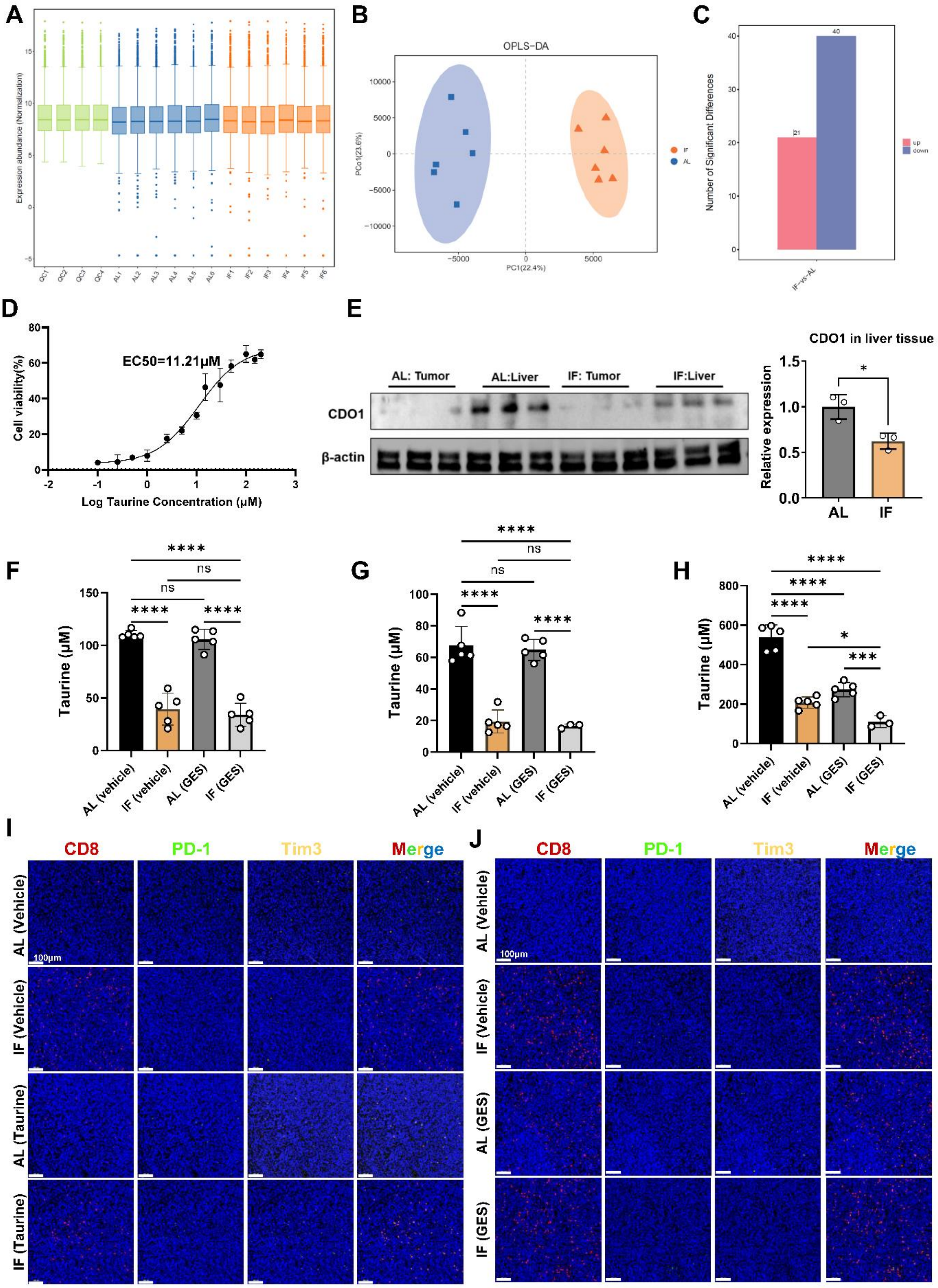

Figure S2. Validation of taurine reduction across systemic and tumor compartments after intermittent fasting.

(A) Quality-control analysis of serum metabolomic profiling.

(B) OPLS-DA score plot showing metabolic separation between AL- and IF-fed tumor-bearing mice.

(C) Numbers of serum metabolic features significantly increased (n = 21) or decreased (n = 40) in IF-fed mice relative to AL-fed mice.

(D) Dose-response analysis of CRC cell viability after taurine treatment. EC50 was calculated by nonlinear regression. n = 3 independent experiments.

(E) Western blot analysis and quantification of hepatic CDO1 expression in AL- and IF-fed mice. n = 3 biological replicates per group. Unpaired t-test.

(F-H) Targeted taurine quantification in serum (F), tumor interstitial fluid (TIF) (G), and tumor tissues (H) from AL- or IF-fed mice treated with vehicle or GES. n = 5 mice per group. One-way ANOVA.

(I) Representative multiplex immunofluorescence images of CD8, PD-1, and TIM-3 in tumor tissues from AL- and IF-fed mice treated with vehicle or taurine.

(J) Representative multiplex immunofluorescence images of CD8, PD-1, and TIM-3 in tumor tissues from AL- and IF-fed mice treated with vehicle or GES. CD8, red; PD-1, green; TIM-3, yellow; nuclei, DAPI (blue). Scale bars, 100 μm.

Data are presented as mean ± SD. *ns, not significant; *P < 0.05, ***P < 0.001, ****P < 0.0001.*

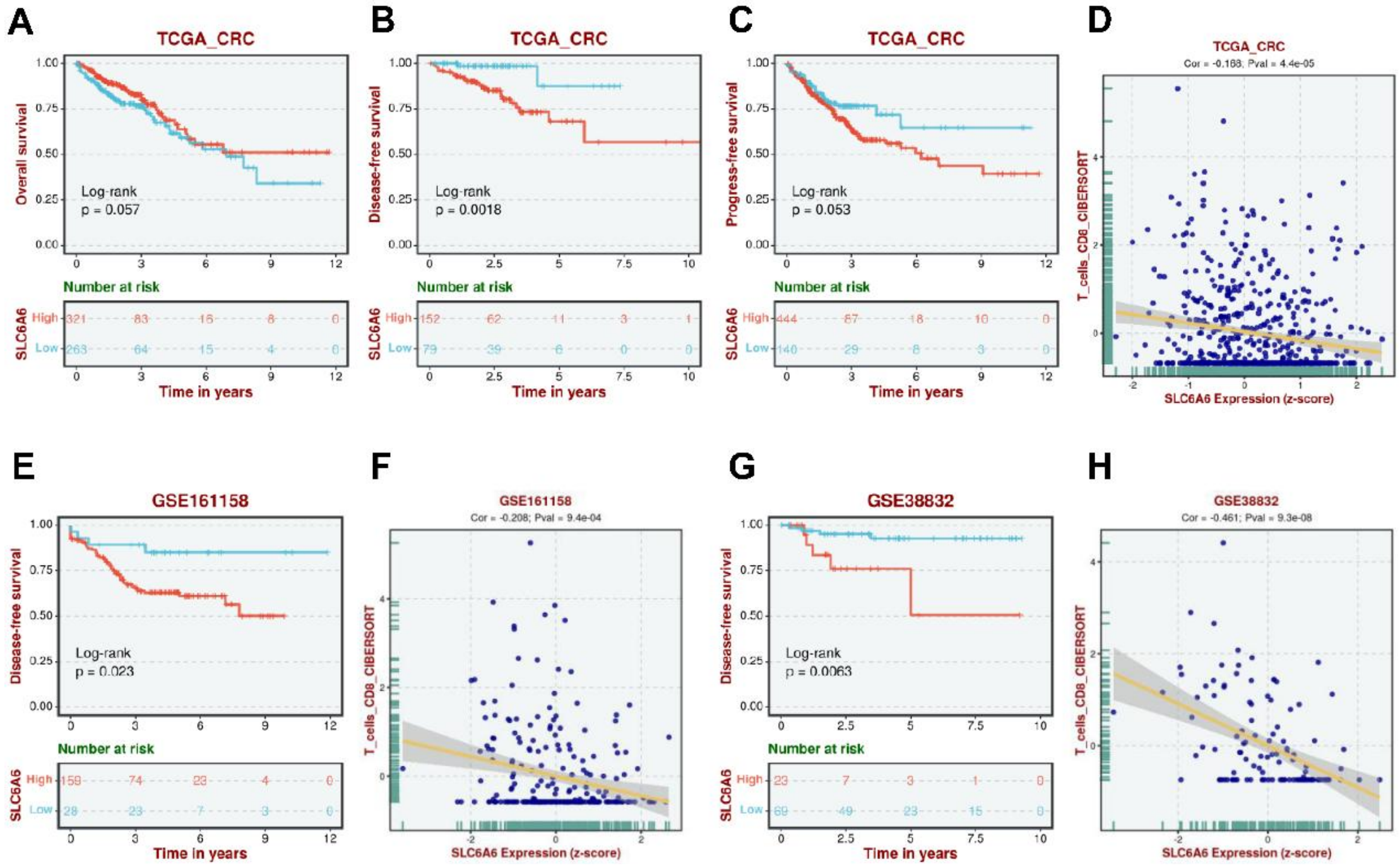

Figure S3. Clinical association of SLC6A6 expression with CRC prognosis and CD8⁺ T-cell infiltration.

(A-C) Kaplan-Meier analyses of overall survival (A), disease-specific survival (B), and progression-free survival (C) according to SLC6A6 expression in the TCGA CRC cohort. Log-rank test.

(D) Correlation between SLC6A6 expression and $CD8^+$ T-cell infiltration in the TCGA CRC cohort. Pearson correlation analysis.

(E, F) Disease-free survival analysis according to SLC6A6 expression (E) and correlation between SLC6A6 expression and $CD8^+$ T-cell infiltration (F) in the GSE161158 cohort. Log-rank test for survival analysis; Pearson correlation analysis for correlation analysis.

(G, H) Disease-free survival analysis according to SLC6A6 expression (G) and correlation between SLC6A6 expression and $CD8^+$ T-cell infiltration (H) in the GSE38832 cohort.

Log-rank test for survival analysis; Pearson correlation analysis for correlation analysis.

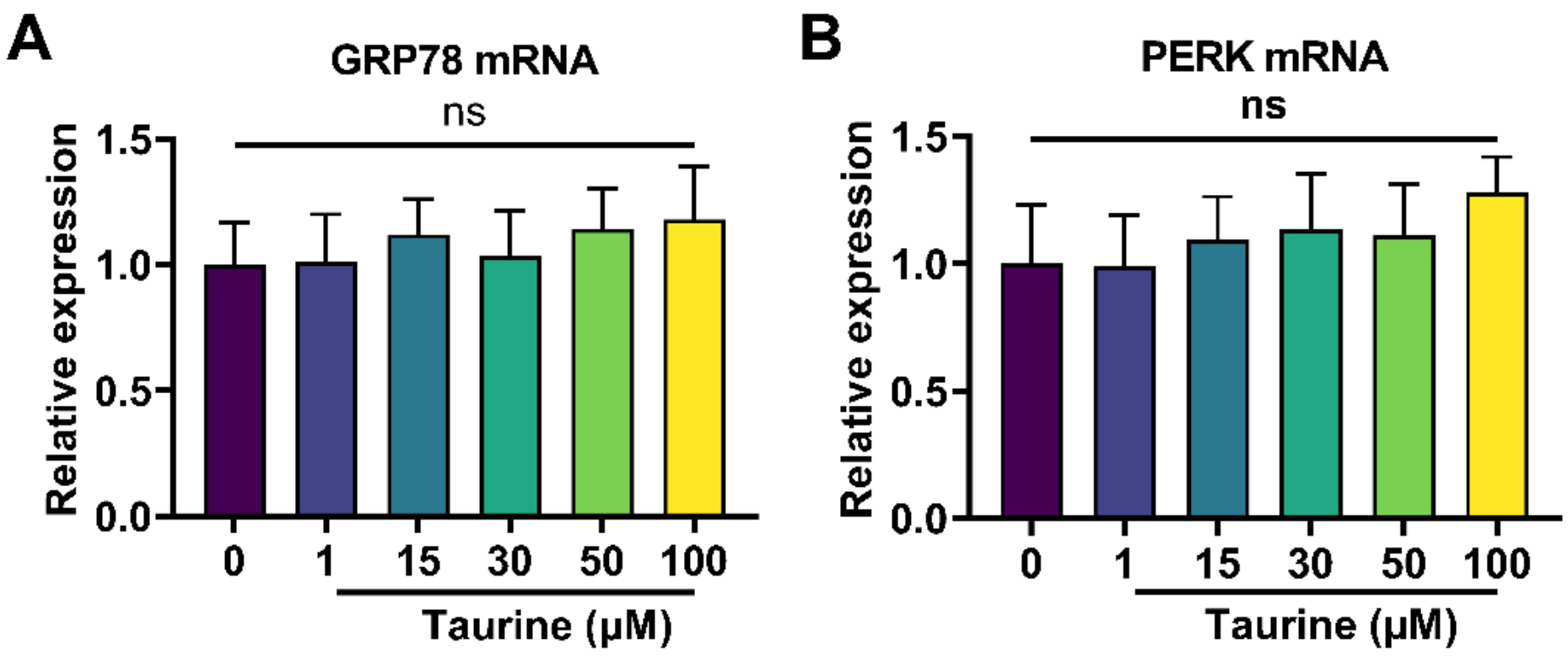

Figure S4. Taurine did not alter GRP78 or PERK mRNA expression.

(A, B) Quantitative PCR analysis of GRP78 (A) and PERK (B) mRNA expression after treatment with increasing concentrations of taurine. n = 3 independent experiments. One-way ANOVA.

Data are presented as mean ± SD. *ns, not significant.*

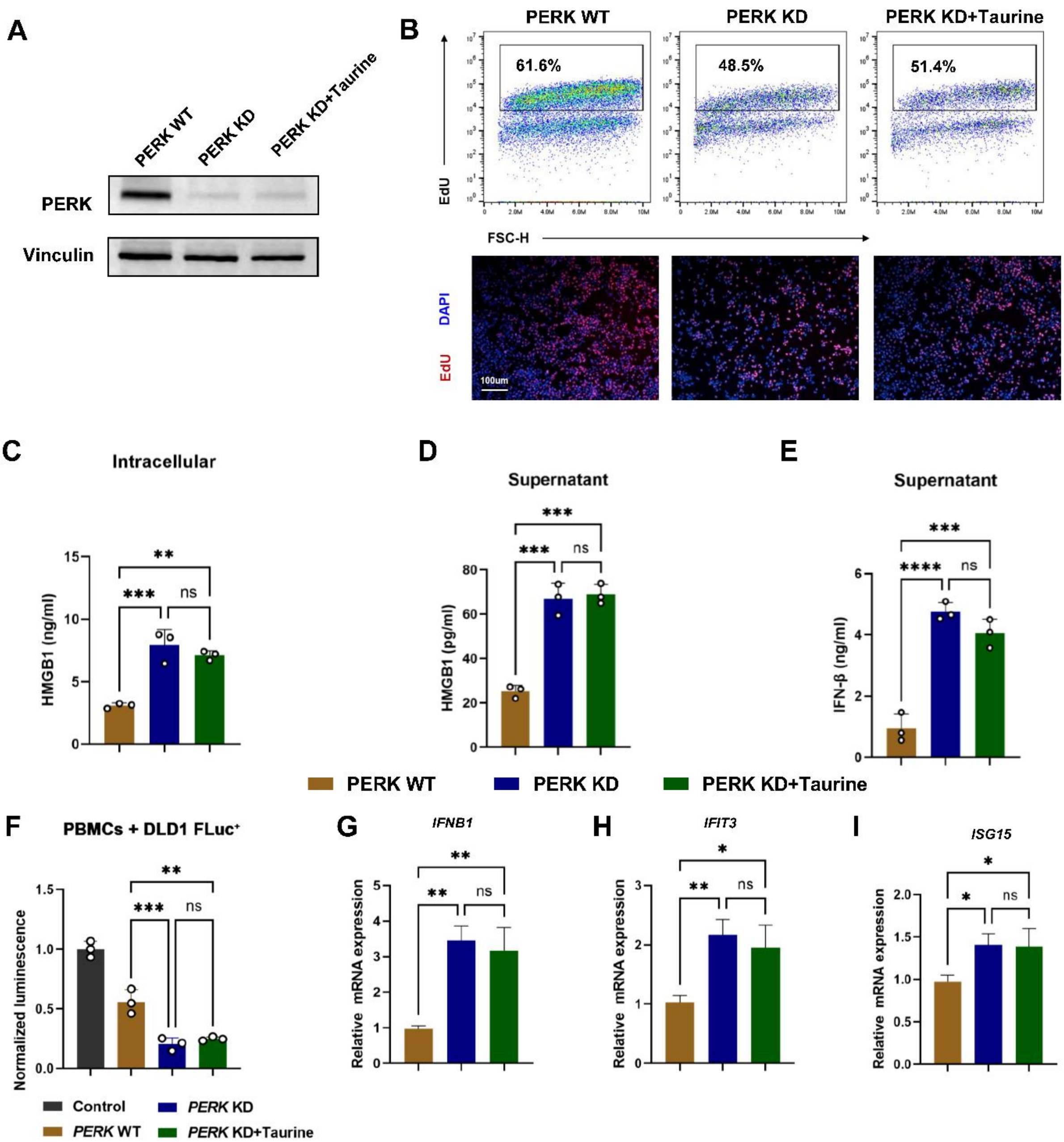

Figure S5. PERK depletion promotes immunogenic stress and immune-mediated tumor-cell killing.

(A) Western blot validation of PERK knockdown in CRC cells. Representative blots from 3 independent experiments are shown.

(B) Representative EdU flow-cytometry plots and EdU immunofluorescence images in PERK WT, PERK KD, and PERK KD plus taurine-treated CRC cells. n = 3 independent experiments.

(C, D) Intracellular HMGB1 (C) and extracellular HMGB1 in culture supernatants (D) from PERK WT, PERK KD, and PERK KD plus taurine-treated cells co-cultured with PBMCs. n = 3 independent experiments. One-way ANOVA.

(E) IFN-β levels in culture supernatants from PERK WT, PERK KD, and PERK KD plus taurine-treated cells co-cultured with PBMCs. n = 3 independent experiments. One-way ANOVA.

(F) PBMC-mediated killing of DLD1-Fluc cells under the indicated conditions. n = 3 independent experiments. One-way ANOVA.

(G-I) Quantitative PCR analysis of *Ifnb1* (G), *Ifit3* (H), and *Isg15* (I) expression in PBMCs which co-cultured with PERK WT, PERK KD, and PERK KD plus taurine-treated cells. n = 3 independent experiments. One-way ANOVA.

Scale bars are shown in the corresponding panels. Data are presented as mean ± SD.

*ns, not significant; *P < 0.05, **P < 0.01, ***P < 0.001, ****P < 0.0001.*

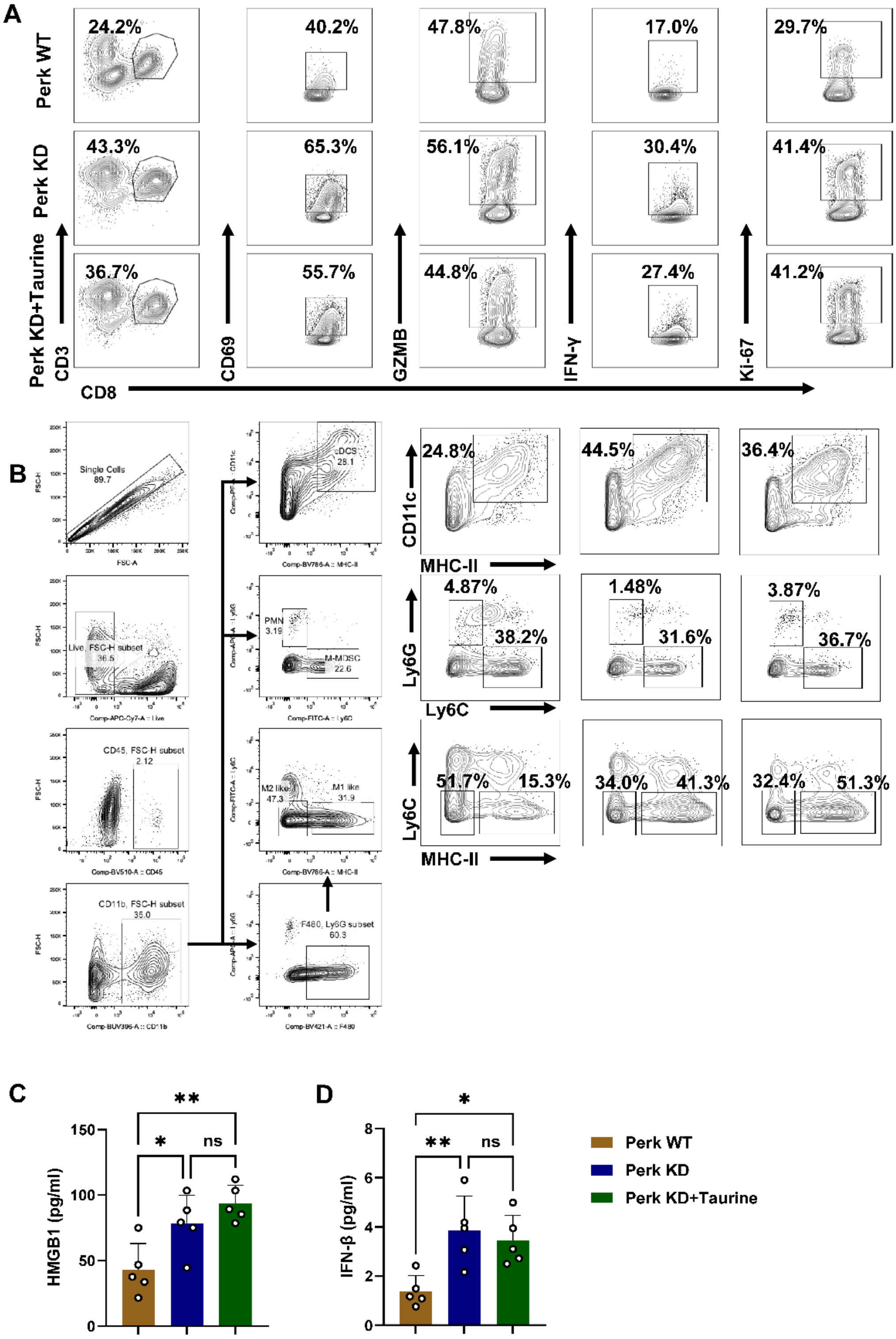

Figure S6. Representative flow-cytometry plots for PERK depletion-induced immune remodeling. (A) Representative flow-cytometry plots showing CD8⁺ T-cell infiltration and activation markers, including CD69, GZMB, IFN-γ, and Ki-67, in PERK WT, PERK KD, and PERK KD plus taurine tumors.

(B) Gating strategy and representative plots for tumor-infiltrating myeloid cells. Myeloid cells were gated as live $CD45^{+}TCR\beta^{-}NKp46^{-}CD19^{-}CD11b^{+}$ cells. cDCs were defined as $CD11c^{+}MHC\text{-}II^{+}$ cells, and activated cDCs as $CD11c^{+}MHC\text{-}II^{+}CD86^{+}$ cells. Monocytic myeloid cells/M-MDSCs were defined as $Ly6C^{+}Ly6G^{-}$ cells, and granulocytic myeloid cells/PMN-MDSCs were defined as $Ly6G^{+}Ly6C^{-}$ cells. Macrophage subsets were analyzed within $F4/80^{+}Ly6G^{-}$ cells; M1-like macrophages were defined as $MHC\text{-}II^{+}Ly6C^{-}CD86^{+}$ cells, and M2-like macrophages as $MHC\text{-}II^{-}Ly6C^{-}$CD86^low/− cells.

(C, D) TIF HMGB1 (C) and IFN-β (D) levels in PERK WT, PERK KD, and PERK KD plus taurine tumor-bearing mice. n = 5 mice per group. One-way ANOVA.

Data are presented as mean ± SD. *ns, not significant; *P < 0.05, **P < 0.01.*

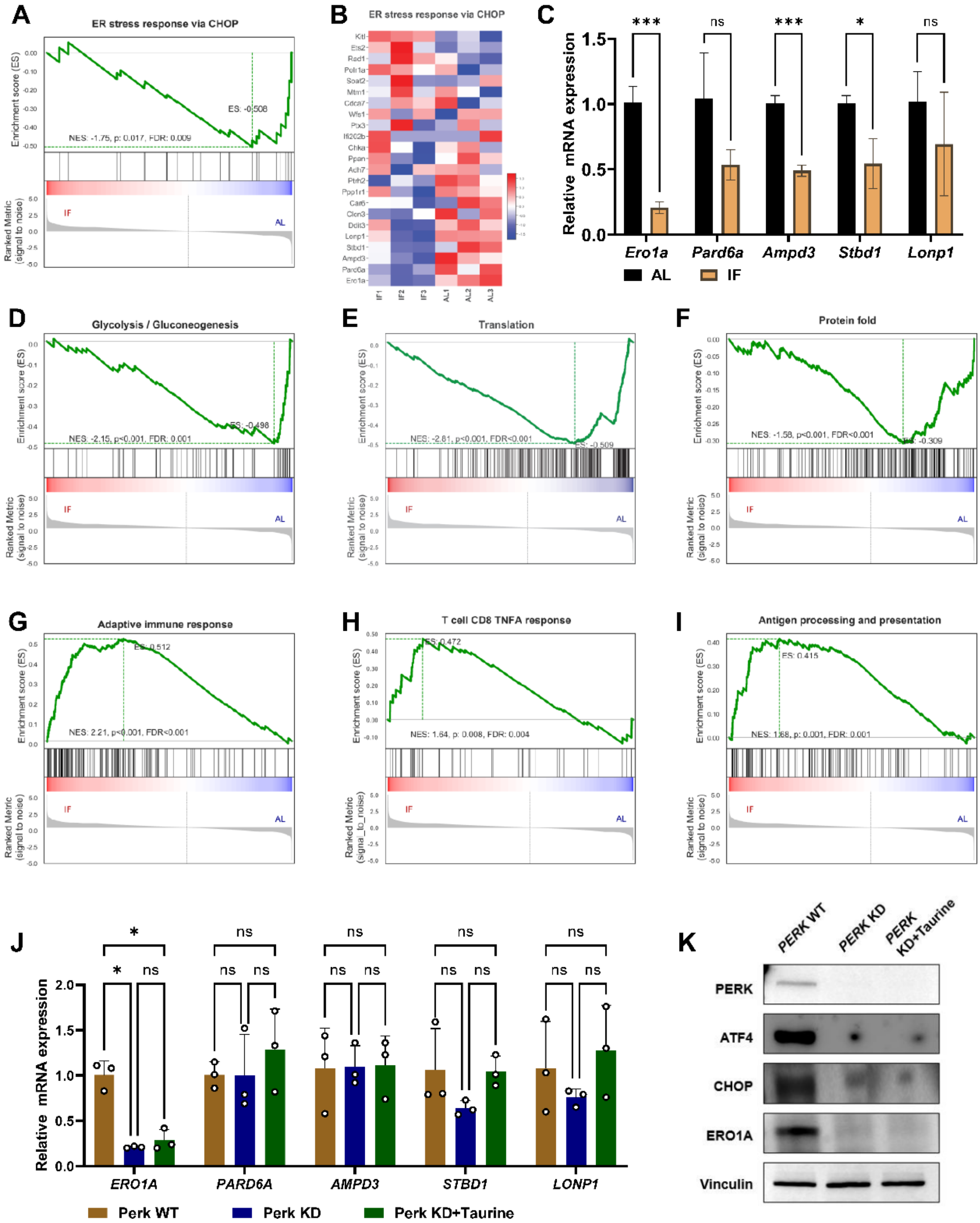

Figure S7. Transcriptomic analyses identify suppression of CHOP-associated ER-stress programs and ERO1A after IF.

(A) GSEA plot showing reduced enrichment of the CHOP-associated ER-stress response program after IF.

(B) Heatmap of CHOP-associated candidate genes in tumors from AL- and IF-fed mice.

(C) Quantitative PCR validation of *Ero1a, Pard6a, Ampd3, Stbd1,* and *Lonp1* expression in AL- and IF-fed tumors. n = 3 biological replicates per group. Unpaired t-test for each gene.

(D-I) GSEA plots showing changes in glycolysis/gluconeogenesis (D), translation (E), protein folding (F), adaptive immune response (G), $CD8^+$ T-cell TNF response (H), and antigen processing and presentation (I) after IF.

(J) Quantitative PCR analysis o*f ERO1A, PARD6A, AMPD3, STBD1,* and *LONP1* expression in Perk WT, Perk KD, and Perk KD plus taurine tumors. n = 3 biological replicates per group. One-way ANOVA for each gene.

(K) Western blot analysis of PERK, ATF4, CHOP, and ERO1A in PERK WT, PERK KD, and PERK KD plus taurine tumors. Representative blots from 3 biological replicates are shown.

Data are presented as mean ± SD. *ns, not significant; *P < 0.05, ***P < 0.001.* NES and FDR values are shown in the corresponding GSEA panels.

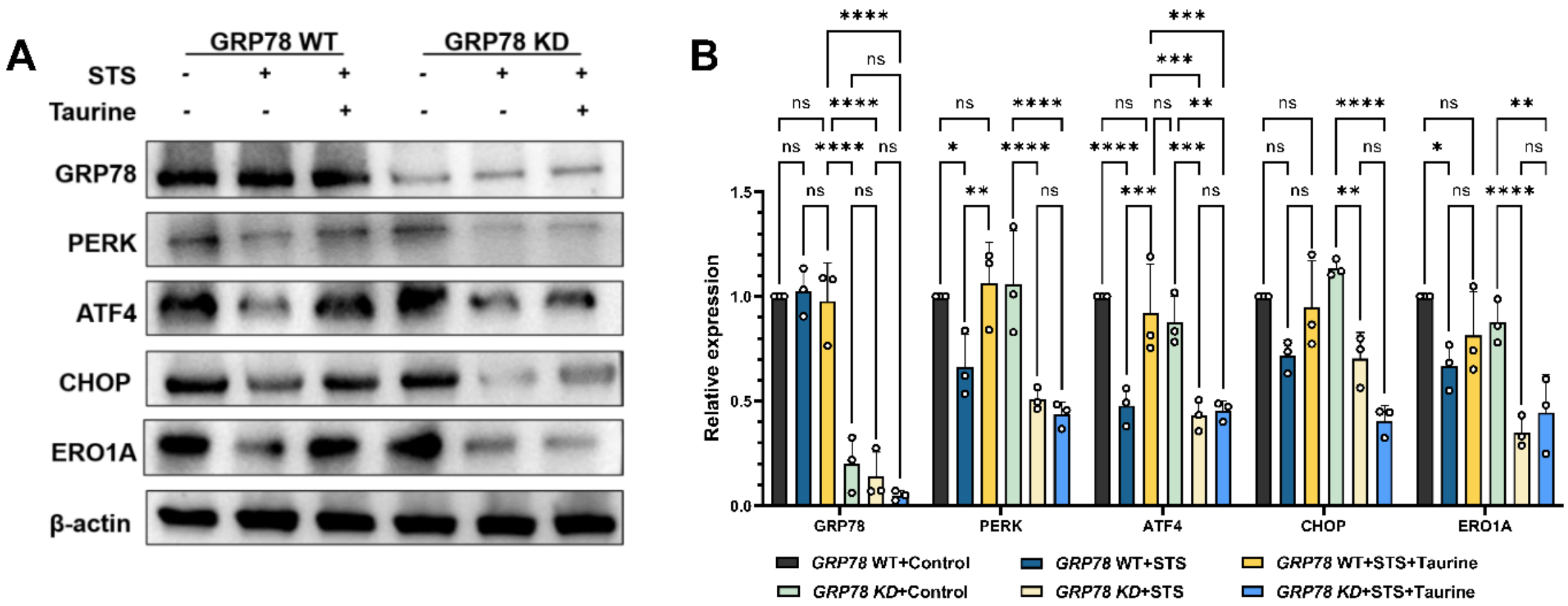

Figure S8. GRP78 is required for taurine-mediated PERK maintenance.

(A) Representative immunoblot analysis of GRP78, PERK, ATF4, CHOP, and ERO1A in GRP78-WT and GRP78-KD CRC cells treated with STS in the presence or absence of taurine. β-Actin was used as the loading control.

(B) Quantification of the immunoblot results. Protein abundance was normalized to β-actin and expressed relative to that in untreated GRP78-WT cells. n = 3 independent experiments. Two-way ANOVA for each protein.

Scale bars are shown in the corresponding panels. Data are presented as mean ± SD. *ns, not significant; *P < 0.05, **P < 0.01, ***P < 0.001, ****P < 0.0001*.

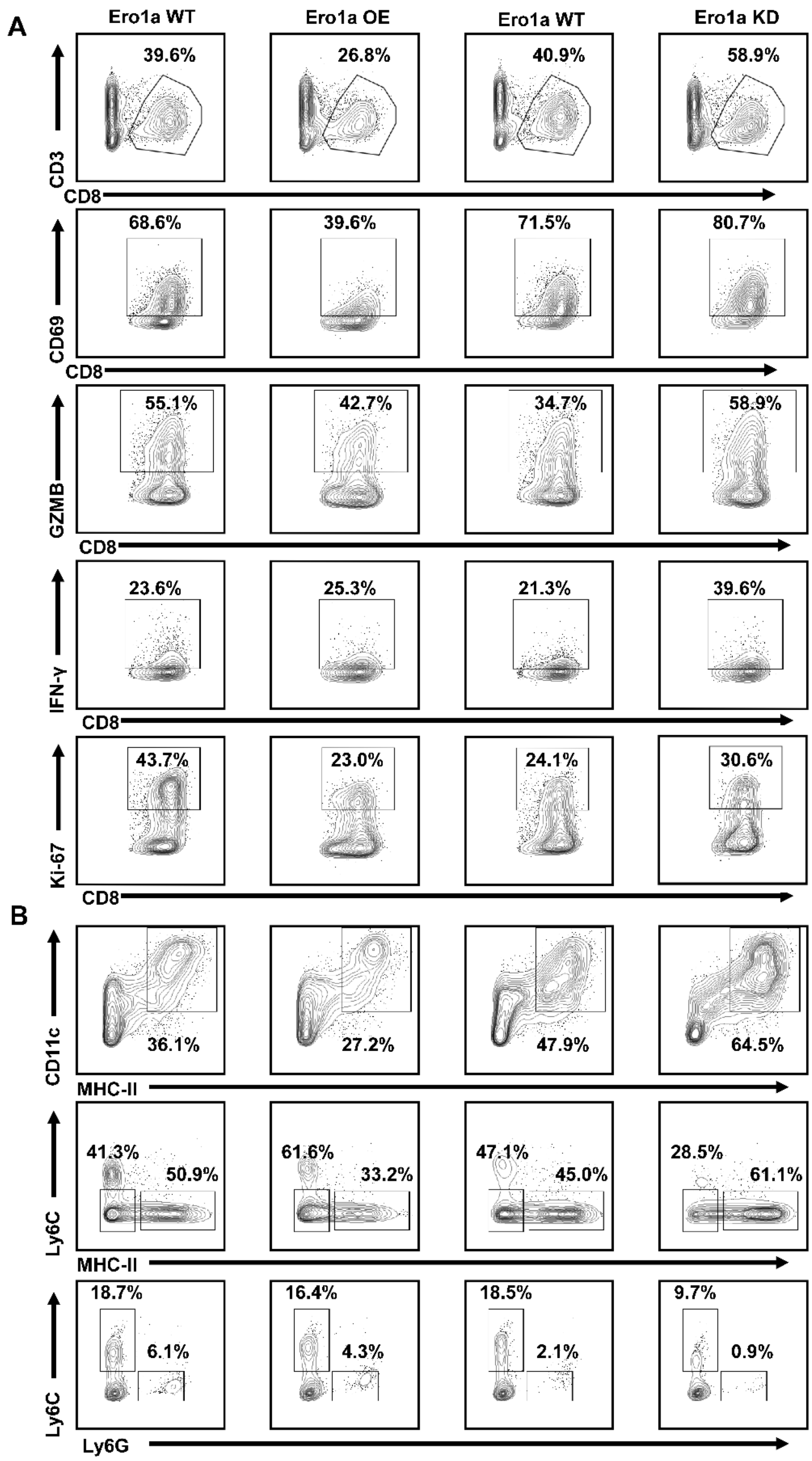

Figure S9. Representative flow-cytometry plots for ERO1A-mediated immune remodeling.

(A) Representative flow-cytometry plots showing CD8^{+} T-cell infiltration and activation markers, including CD69, GZMB, IFN-γ, and Ki-67, in ERO1A WT, ERO1A-overexpressing, and ERO1A-knockdown tumors.

(B) Representative flow-cytometry plots showing tumor-infiltrating myeloid populations in ERO1A WT, ERO1A-overexpressing, and ERO1A-knockdown tumors. cDCs were defined as $CD11c^{+}MHC\text{-}II^{+}$ cells. Monocytic myeloid cells/M-MDSCs were defined as $Ly6C^{+}Ly6G^{-}$ cells, and granulocytic myeloid cells/PMN-MDSCs were defined as $Ly6G^{+}Ly6C^{-}$ cells. Macrophage subsets were analyzed according to MHC-II and Ly6C expression within the myeloid/macrophage gate.

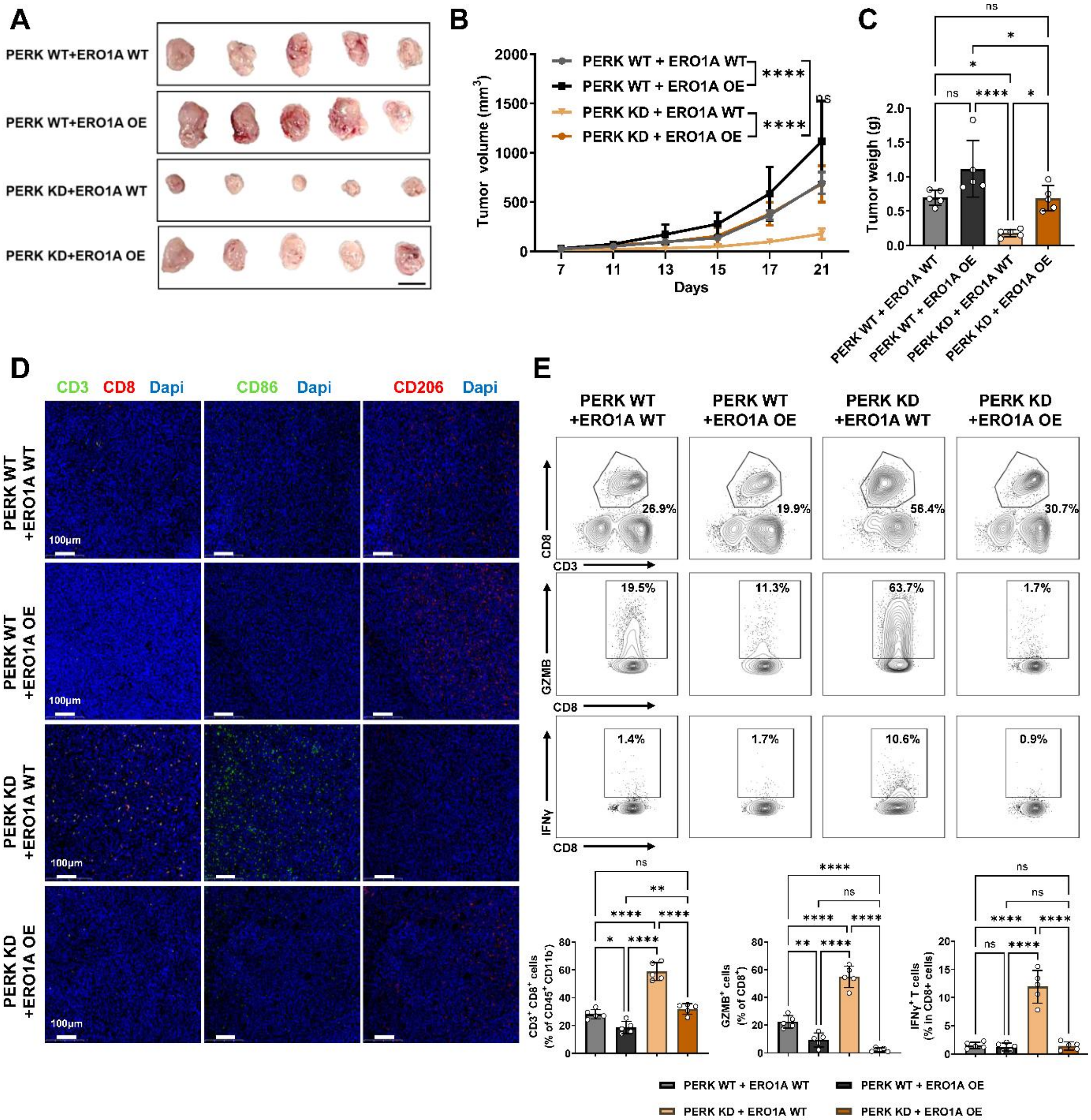

Figure S10. ERO1A overexpression reverses PERK-depletion-mediated tumor suppression.

(A) Representative images of excised tumors derived from PERK-WT or PERK-KD CRC cells with control expression or ERO1A overexpression. Scale bar, 1 cm.

(B) Tumor growth curves for the indicated groups.

(C) Tumor weights measured at the experimental endpoint.

(D) Representative immunofluorescence images of tumor tissues stained for CD3 (green) and CD8 (red), CD86 (green), or CD206 (red), with DAPI nuclear counterstaining (blue). Scale bars, 100 µm.

(E) Representative flow cytometric plots and quantification of intratumoral $CD3^+CD8^+$ cells among $CD45^+CD11b^-$ cells, $GZMB^+$ cells among $CD8^+$ T cells, and IFN-γ^+ cells among $CD8^+$ T cells. For (A–E), n = 5 tumors per group.

Scale bars are shown in the corresponding panels. Data are presented as mean ± SD. *ns, not significant; *P < 0.05, **P < 0.01, ***P < 0.001, ****P < 0.0001.*

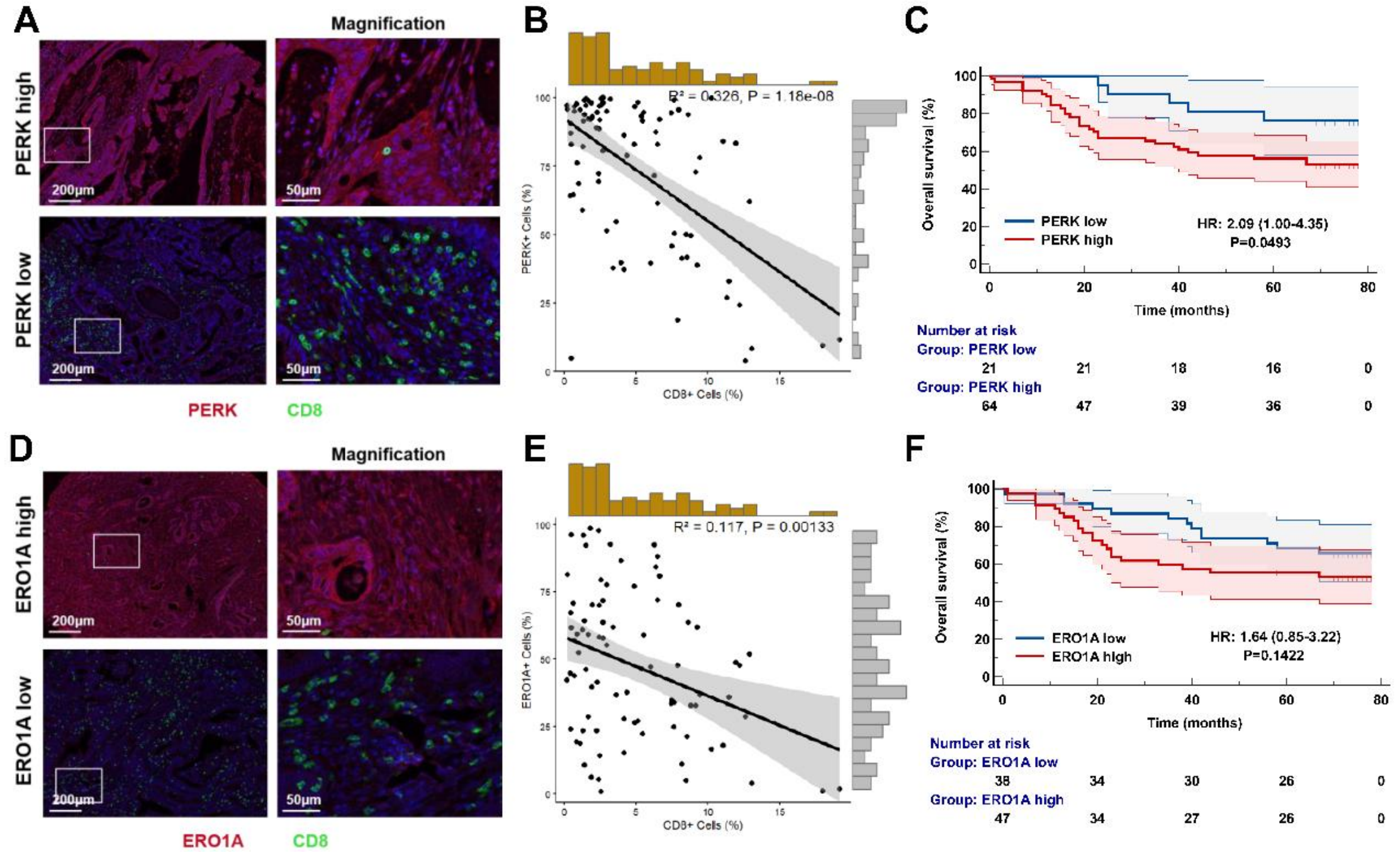

Figure S11. Clinical relevance of PERK and ERO1A in human CRC.

(A) Representative multiplex immunofluorescence images showing PERK and CD8 expression in human CRC tissues with high or low PERK expression.

(B) Correlation between PERK expression and $CD8^+$ T-cell abundance in human CRC tissues. n = 85 cases. Pearson correlation analysis.

(C) Kaplan-Meier analysis of overall survival according to PERK expression. n = 85 patients. Log-rank test.

(D) Representative multiplex immunofluorescence images showing ERO1A and CD8 expression in human CRC tissues with high or low ERO1A expression.

(E) Correlation between ERO1A expression and $CD8^+$ T-cell abundance in human CRC tissues. n = 85 cases. Pearson correlation analysis.

(F) Kaplan-Meier analysis of overall survival according to ERO1A expression. n = 85 patients. Log-rank test.

Scale bars are shown in the corresponding panels.

Table S1. Baseline characteristics of cohort 1

**Characteristic**	**Overall (N=85)**
**Total patients**	85
**Age, years, median [IQR]**	65.0 [57.0, 77.0]
**Sex**	
Male	44 (51.8%)
Female	41 (48.2%)
**Vital status**	
Alive	50 (58.8%)
Dead	35 (41.2%)
**Follow-up/survival time, months, median [IQR]**	70.0 [23.0, 75.0]
**Tumor characteristics**	
**Tumor site**	
Right colon	32 (37.6%)
Left colon/sigmoid	39 (45.9%)
Transverse/flexure colon	12 (14.1%)
Colon, not otherwise specified	2 (2.4%)
**Histology**	
Adenocarcinoma	71 (83.5%)
Mucinous adenocarcinoma/component	6 (7.1%)
Tubular adenocarcinoma	4 (4.7%)
Tubulopapillary adenocarcinoma	2 (2.4%)
Signet-ring cell carcinoma	1 (1.2%)
Adenocarcinoma with an undifferentiated component	1 (1.2%)
**Pathological grade**	
I	2 (2.4%)
I-II	16 (18.8%)
II	54 (63.5%)
II-III	7 (8.2%)
III	5 (5.9%)
III-IV	1 (1.2%)
**Maximum tumor diameter, cm, median [IQR]**	5.0 [4.0, 6.0]
**Distant metastasis**	
No	80 (94.1%)
Yes	5 (5.9%)
**Pathological stage**	
**T stage**	
T1	1 (1.2%)
T2	9 (10.6%)
T3	47 (55.3%)
T4a	23 (27.1%)
T4b	5 (5.9%)
**N stage**	
N0	53 (62.4%)
N1a	8 (9.4%)
N1b	15 (17.6%)

N2a	4 (4.7%)
N2b	5 (5.9%)
**M stage**	
M0	80 (94.1%)
M1a	3 (3.5%)
M1b	2 (2.4%)
**Lymph node metastasis**	
Negative	52 (61.2%)
Positive	32 (37.6%)
Missing	1 (1.2%)
**Lymph nodes yield, median [IQR]**	8.0 [5.0, 11.0]
**Positive lymph nodes, median [IQR]**	0.0 [0.0, 2.0]
**AJCC 7th clinical stage**	
I	10 (11.8%)
IIA	33 (38.8%)
IIB	7 (8.2%)
IIC	1 (1.2%)
IIIB	19 (22.4%)
IIIC	10 (11.8%)
IVA	3 (3.5%)
IVB	2 (2.4%)
**IHC marker status**	
**PERK**	
Low expression	23 (27.1%)
High expression	62 (72.9%)
**ERO1A**	
Low expression	38 (44.7%)
High expression	47 (55.3%)
**SLC6A6**	
Low expression	23 (27.1%)
High expression	62 (72.9%)
**CD8**	
Low expression	56 (65.9%)
High expression	29 (34.1%)

Table S2. Baseline characteristics of cohort 2

Characteristic	Overall (N=23)
**Patient characteristics**	
No. of patients	23 (100%)
**Age, years, median [IQR]**	67 (49-72)
Sex, male	16 (69.6%)
**Adenoma characteristics**	
No. of adenoma samples	23 (100%)
Adenoma size, cm	1.2 (0.8-1.5)
Histologic type	
Tubular adenoma, n (%)	3 (13.1%)
Tubulovillous adenoma, n (%)	7 (30.4%)
Villous adenoma, n (%)	13 (56.5%)
Dysplasia grade	
Low-grade dysplasia, n (%)	7 (30.4%)
High-grade dysplasia, n (%)	16 (69.6%)
**Carcinoma characteristics**	
No. of carcinoma samples	23 (100%)
Tumor size, cm	3.0 (2.5-5.0)
Histologic type	
Adenocarcinoma, n (%)	23 (100%)
Other type, n (%)	0 (0%)
Differentiation	
Well differentiated, n (%)	11 (47.8%)
Moderately differentiated, n (%)	10 (43.5%)
Poorly differentiated, n (%)	2 (8.7%)
TNM stage	
Stage I, n (%)	6 (26.1%)
Stage II, n (%)	11 (47.8%)
Stage III, n (%)	6 (26.1%)
Stage IV, n (%)	0 (0%)

Table S3. Key Resources

REAGENT or RESOURCE	SOURCE	IDENTIFIER
**Antibodies**		
Anti-mouse CD8α (in vivo)	TargetMol	Cat# T9901A-629; Clone: YTS 169.4
Mouse IgG2a kappa isotype control antibody (in vivo)	TargetMol	Cat# T78337
Anti-mouse PD-1 antibody (in vivo)	TargetMol	Cat# T78269; Clone: RMP1-14
CoraLite® Plus 750-conjugated calreticulin recombinant monoclonal antibody	Proteintech	Cat# CL750-80017
Anti-SLC6A6	Abcam	Cat# ab236898
Anti-CD8	Abcam	Cat# ab217344
Anti-EIF2AK3 (PERK)	Proteintech	Cat# 24390-1-AP
Anti-Mouse CD45 BUV395	BD Biosciences	Cat#564279
Anti-Mouse CD11b PE-Cyanine7	Thermo Fisher Scientific	Cat#25-0112-82
Anti-mouse CD3 Brilliant Violet 421	Biolegend	Cat#100227
Anti-mouse CD8 FITC	Biolegend	Cat#100705
Anti-mouse IFN-γ APC	Biolegend	Cat#505810
Anti-mouse Granzyme B PE	Biolegend	Cat#168804
Anti-mouse CD279 (PD-1) Brilliant Violet 605	Biolegend	Cat#135219
Anti-Mouse CD45 BV510	Biolegend	Cat#157219
Anti-mouse CD11b PerCP/Cyanine5.5	Biolegend	Cat#101227
Anti-mouse CD69 PE/Cyanine7	Biolegend	Cat#310912
Anti-mouse CD8 BUV395	Thermo Fisher Scientific	Cat#363-0081-82
Anti-mouse Ki67 FITC	Biolegend	Cat#652410

Anti-Mouse CD45 BV510	BD Biosciences	Cat#157219
Anti-Mouse CD11b Spark PLUS UV395	Biolegend	Cat#101240
Anti-mouse Ly-6G APC	Biolegend	Cat#127614
Anti-Mouse Ly-6C FITC	Biolegend	Cat#128006
Anti-Mouse F4/80 BV421	Biolegend	Cat#123137
Anti-Mouse CD11c PE	Biolegend	Cat#117308
Anti-Mouse I-A/I-E BV786	BD Biosciences	Cat#742894
Anti-EIF2AK3	Abmart	Cat#MA8131
Anti-EIF2AK3	Proteintech	Cat# 68482-1-Ig
Anti-GRP78 (Bip/HSPA5)	Abmart	Cat#TB5893
Anti-GRP78 (Bip/HSPA5)	Proteintech	Cat#11587-1-AP
Anti-ERO1A	Proteintech	Cat#12007-1-AP
Anti-ATF-4	Proteintech	Cat#10835-1-AP
Anti-ubiquitin	Proteintech	Cat#10201-2-AP
Anti-IRE1α	Cell sginalling	Cat#3294
Anti-ATF-6	Cell sginalling	Cat#65880
Anti-CDO1	Proteintech	Cat#12589-1-AP
Anti-Vinculin	Proteintech	Cat#26520-1-AP
Anti-β-actin	Proteintech	Cat#66009-1-Ig
Anti-SEC61B	Proteintech	Cat#15087-1-AP
HRP-conjugated Goat Anti-Rabbit IgG(H+L)	Proteintech	Cat#SA00001-2
HRP-conjugated Goat Anti-Mouse IgG(H+L)	Proteintech	Cat#SA00001-1
Anti-rabbit IgG, HRP-linked Antibody	Cell Signaling Technology	Cat#7074
Anti-mouse IgG, HRP-linked Antibody	Cell Signaling Technology	Cat#7076
Anti-mouse IgG (H+L), Alexa Fluor® 488 Conjugate	Cell Signaling Technology	Cat#4408
Anti-mouse IgG (H+L), Alexa Fluor® 568 Conjugate	Cell Signaling Technology	Cat#39572

**Experimental Models: Animal and Cell lines**		
C57BL/6JGpt mice	GemPharmatech Co., Ltd.	Cat# N000013
BALB/cJGpt mice	GemPharmatech Co., Ltd.	Cat# N000020
$Apc^{Min/+}$ mice	GemPharmatech Co., Ltd.	Cat# T001457
DLD-1	CAS Center for Excellence in Molecular Cell Science	Cat# TCHu134
MC38	CAS Center for Excellence in Molecular Cell Science	Cat# SCSP-5431
CT26.WT	CAS Center for Excellence in Molecular Cell Science	Cat# TCM37
**Chemicals**		
Dulbecco's modified Eagle's medium (DMEM)	MeilunBio	Cat# MA0212
Fetal bovine serum (FBS)	MeilunBio	Cat# PWL217
Antibiotics	MeilunBio	Cat# MA0345
Glucose-free DMEM	MeilunBio	Cat# MA0581
Glucose	MeilunBio	Cat# BGC001
Taurine	TargetMol	Cat# T0022
Z-VAD-FMK	TargetMol	Cat# T7020
Necrostatin-1	TargetMol	Cat# T1847
Ferrostatin I	TargetMol	Cat# T6500
Dapansutrile	TargetMol	Cat# T15052
Chloroquine	TargetMol	Cat# T8689
AZ9482	TargetMol	Cat# T22264
Human recombinant IL-2	MeilunBio	Cat# MB5951
**Critical Commercial Assays**		
Mycoplasma detection kit	Yeasen	Cat# 40612ES25
Ficoll-Paque Premium	GE Healthcare	Cat# 29264741-3-2
ImmunoCult™ Human CD3/CD28 T Cell Activator	STEMCELL Technologies	Cat# 100-0784
ImmunoCult™-XF	STEMCELL Technologies	Cat# 100-0956
Foxp3 / Transcription Factor Staining Buffer Set	Thermo Fisher Scientific	Cat# 00-5523-00
Fixable Viability Dye eFluor™ 780	Thermo Fisher Scientific	Cat# 65-0865-14
Fc blocker	BD Biosciences	Cat# 553142
Cell Stimulation Cocktail (plus protein transport inhibitors)	Thermo Fisher Scientific	Cat# 00-4975

Annexin V-PE/7-AAD Cell Apoptosis Detection Kit	MeilunBio	Cat# MA0429
ER-Tracker Red	Beyotime	Cat# C1041S
EdU Cell Proliferation Kit with Alexa Fluor 555	MeilunBio	Cat# MA0425-1
Taurine Assay Kit	Absin	Cat# abs580222
BCA Protein Assay Kit	Beyotime	Cat# P0009
Mouse HMGB1 ELISA Kit	Thermo Fisher Scientific	Cat# EEL102
Mouse IFN-β ELISA Kit	R&D Systems	Cat# DIFNB0
Human HMGB1 ELISA Kit	Thermo Fisher Scientific	Cat# EEL047
Human IFN-β ELISA Kit	R&D Systems	Cat# 42400-1
Firefly Luciferase Reporter Gene Assay Kit	Beyotime	Cat# RG005
Cell Counting Kit-8	Beyotime	Cat# C0037
Opal 7-Color Manual IHC Kit	PerkinElmer	Cat# NEL811001KT
TRIzol	Thermo Fisher Scientific	Cat# 15596026CN
V RT reagent Kit	Takara	Cat# RR047B
TB Green® Fast qPCR Mix	Takara	Cat# RR430B
M-PER™ Mammalian Protein Extraction Reagent	Thermo Fisher Scientific	Cat# 78501
PVDF membrane	Merck Millipore	Cat# SEQ85R
ECL luminescence reagent	MeilunBio	Cat# MA0186-2
DAPI Solution	BD Biosciences	Cat# 564907
**Oligonucleotides and Primers**		
EIF2AK3 Forward	Sangon Biotech	ACGATGAGACAGAGTTGCGAC
EIF2AK3 Reverse	Sangon Biotech	ATCCAAGGCAGCAATTCTCCC
HSPA5 Forward	Sangon Biotech	CATCACGCCGTCCTATGTCG
HSPA5 Reverse	Sangon Biotech	CGTCAAAGACCGTGTTCTCG
SLC6A6 Forward	Sangon Biotech	GGAGAAGTGGTCTAGCAAGATCG
SLC6A6 Reverse	Sangon Biotech	AGAAACGCACCTCCACCATTC
IFNB1 Forward	Sangon Biotech	ATGACCAACAAGTGTCTCCTCC
IFNB1 Reverse	Sangon Biotech	GGAATCCAAGCAAGTTGTAGCTC
IFIT3 Forward	Sangon Biotech	TCAGAAGTCTAGTCACTTGGGG
IFIT3 Reverse	Sangon Biotech	ACACCTTCGCCCTTTCATTTC
ISG15 Forward	Sangon Biotech	CGCAGATCACCCAGAAGATCG

ISG15 Reverse	Sangon Biotech	TTCGTCGCATTTGTCCACCA
ACTB Reverse	Sangon Biotech	ACCGGGCATAGTGGTTGGA
ACTB Forward	Sangon Biotech	ATGGTACACGGTTCTCAACATC
**shRNA Target Sequence**		
shEIF2AK3	Shanghai Genechem Co., Ltd.	GCACACAGATTACAGTCAGAT
shHSPA5	Shanghai Genechem Co., Ltd.	GAAATCGAAAGGATGGTTAAT
shEif2ak3	Shanghai Genechem Co., Ltd.	GCCACTTTGAACTTCGGTATA
shEro1a	Shanghai Genechem Co., Ltd.	GTCCTAAGCCAAACGTTTATA
